# Long-term annual soil nitrogen surplus across Europe (1850–2019)

**DOI:** 10.1038/s41597-022-01693-9

**Published:** 2022-10-10

**Authors:** Masooma Batool, Fanny J. Sarrazin, Sabine Attinger, Nandita B. Basu, Kimberly Van Meter, Rohini Kumar

**Affiliations:** 1 UFZ-Helmholtz Centre for Environmental Research, Department of Computational Hydrosystems, Leipzig, Germany; 2 Institute of Environmental Science and Geography, University of Potsdam, Potsdam, Germany; 3 Department of Civil and Environmental Engineering, University of Waterloo, Waterloo, ON Canada; 4 Department of Earth and Environmental Sciences, University of Waterloo, Waterloo, ON Canada; 5 Water Institute, University of Waterloo, Waterloo, ON Canada; 6 Department of Geography, The Pennsylvania State University, University Park, PA USA; 7 Earth and Environmental Systems Institute, The Pennsylvania State University, University Park, PA USA

**Keywords:** Hydrology, Biogeochemistry

## Abstract

Worldwide surface waters suffer from the presence of nitrogen (N) compounds causing eutrophication and deterioration of the water quality. Despite many Europe-wide legislation’s, we still observe high N levels across many water bodies in Europe. Information on long-term annual soil N surplus is needed to better understand these N levels and inform future management strategies. Here, we reconstructed and analysed the annual long-term N surplus for both agricultural and non-agricultural soils across Europe at a 5 arcmin (≈10 km at the equator) spatial resolution for more than a century (1850–2019). The dataset consists of 16 N surplus estimates that account for the uncertainties resulting from input data sources and methodological choices in major components of the N surplus. We documented the consistency and plausibility of our estimates by comparing them with previous studies and discussed about possible avenues for further improvements. Importantly, our dataset offers the flexibility of aggregating the N surplus at any spatial scale of relevance to support water and land management strategies.

## Abstract (summary)

**Table:** 

Measurement(s)	Soil nitrogen surplus
Technology Type(s)	digital curation, data aggregation
Sample Characteristic - Environment	agricultural and non-agricultural soil systems
Sample Characteristic - Location	Europe

## Background & Summary

Worldwide, surface waters suffer from high concentration of nitrogen (N) compounds, mainly due to large usages of agrochemicals (inorganic fertilizers)^1,2^ and deposition of atmospheric N originating from fossil fuel combustion^3–5^. This has led to the deterioration of the surface and subsurface water quality, causing blooms of harmful algae and threatening aquatic ecosystems and human health when N compounds are present in drinking water^6–9^. To address these N related problems, water quality mitigation measures were implemented, in particular in Europe following the EU Water Framework Directive (Directive2000/60/EC). Despite such measures, we still observe nitrate levels above the regulatory threshold in several water-bodies across Europe^10,11^. One of the reasons behind such failure lies in the presence of N legacies, as N levels can depend not only on the current N inputs to the landscape, but also on the past N inputs that have accumulated through time in the soil zone and the groundwater in so-called “legacy stores”^12,13^. These legacies are largely responsible for time lags between the implementation of water quality measures and the water quality response^14,15^.

Therefore, information on long-term annual N budgets at relevant scales for water and land management is crucial to better understand N legacies and inform future (land and water) management strategies^16^. Existing datasets covering the entire European domain are either based on the N land budget, which is defined as the difference between N inputs (mineral fertilizer, manure, atmospheric deposition, biological N fixation) and N outputs (N from harvested crops and grass removal)^11^, or the soil surface budget which further excludes gaseous losses during manure management and storage^17^. These datasets provide annual N budgets either for limited time periods or at a coarse spatial resolution and they focus on agricultural soils (cropland and/or pasture) only. In particular, the FAO provides global datasets for soil nutrient budgets at country-level for cropland^18^ and for different underlying components of the agricultural N budget since 1961. More recently, Zhang *et al*.^5^ provides estimates for different components of the N budget for cropland, which were based on 13 existing long-term databases covering 115 countries from 1961 to 2015. Further, N budget datasets were developed for European countries specifically, either with respect to total agricultural lands (Eurostat^19^; Leip *et al*.^20^) or to croplands only (Einarsson *et al*.^21^). Moreover, efforts have been expanded to provide N budgets at sub-national levels for individual countries, such as, Denmark^22^, United Kingdom^23^, France^24^, Poland^25^ and Germany^26^. Notably, uncertainty estimation is also currently not a common practice in N budget construction^17^, although uncertainties in existing N datasets are understood to be large^5^. Uncertainties in the N surplus budget, for example, can stem from a limited knowledge on fertilizer and manure application rates, distinction of their applications among croplands and pasture areas, specifications of livestock and grassland (N) productions, among others^5,27,28^.

To overcome these challenges, we provide a dataset of annual long-term N soil surface budget (termed hereafter as “N surplus”) from both agricultural (cropland and pasture) and non-agricultural soils across Europe at a spatial resolution of 5 arcmin (1/12°; ≈10 km at the equator) over more than a century (1850–2019), while explicitly accounting for uncertainties resulting from input data sources and methodological choices in major components of the N surplus (i.e., fertilizer, manure and N removal rates). This newly developed dataset combines information at different levels (in particular country and grid level) to construct the different N surplus components. We assessed the importance of having a long-term dataset of N surplus, as the magnitude of the N surplus has changed tremendously over the last 100 years. Moreover, we show the relevance of considering N surplus over non-agricultural areas, since these areas account for 30% of the total N surplus on average across the study area of Europe. Our gridded database also offers the flexibility of aggregating N surplus at any spatial scale of relevance for the design of land and management strategies. Importantly, we demonstrate the plausibility and consistency of our N surplus estimates by comparing them with existing N budgets datasets^11,20,22–26,29,30^. We then discuss about possible avenues towards a more comprehensive uncertainty characterization in N surplus. In this respect, our reconstruction methodology, described in detail below, paves the way for the exploration of further alternative assumptions in N surplus.

## Methods

**Fig. 1:**
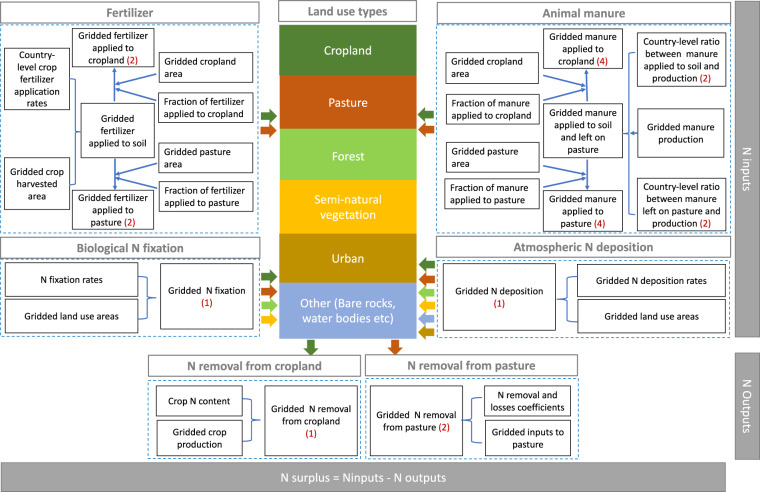
Workflow for constructing the long-term annual dataset of N surplus during the period 1850–2019. The number in brackets with red colors present different combination of datasets that we used to account for the uncertainties, resulting in 16 N surplus estimates.

This section details our methodology for reconstructing the long-term annual time series of the land use and the individual components of the N surplus at gridded level (5 arcmin) over the time period 1850–2019 (see detailed workflow in Fig. 1). We compiled and harmonized a range of databases available for different time periods (i.e., for the periods 1850–1960 and 1961–2019, and for the year 2000), at different frequencies (i.e., yearly, decadal, snapshots) and at different spatial resolutions (i.e, global trends, country-level values, gridded values) following the harmonization methodologies from previous studies^1,16,31,32^. The data sources used in this study are listed in Table 1. Importantly, we accounted for the uncertainties resulting from the differences in underlying data sources and methodological choices in major components of the N surplus. To this end, we constructed 16 gridded time-series of N surplus estimates by combining two estimates for fertilizer, four estimates for animal manure, and two estimates for the N removal from pastures.

**Table 1:** Datasets used in this study for the reconstruction of N surplus.

Datasets	Variables	Spatial feature			Temporal feature		Ref
		Extent	Level	Resolution	Extent	Resolution	
Ramankutty *et al*.^34^	Land use (cropland and pasture area)	Global	Grid	5′ × 5′	2000	Snapshot	^34^
Monfreda *et al*.^35^	Crop specific harvested area and production	Global	Grid	5′ × 5′	2000	Snapshot	^35^
HYDE	Land use (cropland and pasture area)^a^	Global	Grid	5′ × 5′	1700–2017	Decadal or Annual^b^	^40^
Global Land Cover (GLC)	Land use (forest, semi-natural vegetation, urban, non-vegetation area)	Global	Grid	5′ × 5′	2000	Snapshot	^39^
input4MIPS	N atmospheric deposition	Global	Grid	1.9° × 2.5°	1850–2014	Monthly	^53^
Zhang *et al*.^29^	Animal manure production	Global	Grid	5′ × 5′	1850–2014	Annual	^29^
Holland *et al*.^42^	Fertilizer production	Global	Country	—	1925–1960	Annual	^42^
FAOSTAT	Land use (cropland, pasture), fertilizer application, animal manure, crop harvested area and production	Global	Country	—	1961–2019	Annual	^38^
IFA	Fertilizer application amounts for different crop categories and pasture	Global	Country^c^	—	2014/2015	Snapshot	^40^
Bayliss-Smith and Wanmali (1984) as cited in Our World in Data (OWD)	Wheat yield	Global	Country	—	1850–1960	Snapshots^d^	^57,58^
Einarsson *et al*.^21^	Estimates for fodder crops (area, production), fertilizer, manure	Region	Country	—	1961–2019	Annual	^21^

^a^According to the definition used in our study, pasture corresponds to the land under permanent meadow and pasture. Therefore, we used the category “grazing” of the HYDE dataset to derive the pasture areas in our study.

^b^Decadal for the period 1700–2000 and annual for the period 2000–2016.

^c^IFA covers estimates for 28 countries (considering European Union (EU-28) as a single country).

^d^OWD provide wheat yield for the years 1850, 1909, 1934, 1960.

In particular, we adopted country-level data from FAOSTAT (Food and Agriculture Organization Corporate Statistical Database)^33^ that provide the longest data record across a range of variables such as mineral fertilizer, animal manure, crop production and crop harvested area with global coverage during 1961–2019. Furthermore, we utilized the recent country-level dataset of Einarsson *et al*.^21^ available for the period 1961–2019 for animal manure, distribution of fertilizer and manure to cropland and pasture, fodder crop specific harvested areas and fodder crop production. FAOSTAT includes data for total agricultural areas based on national statistics, whereas, Einarsson *et al*.^21^ derives estimates for croplands by combining information from FAOSTAT, Eurostat and several national datasets for European countries. We downscaled the country-level estimates using, for most variables, the spatial variability in land use areas (Ramankutty *et al*.^34^ dataset), and crop specific harvested areas and crop production (Monfreda *et al*.^35^ dataset) for the year 2000. We used consistent grid level datasets^34,35^ to avoid a mismatch at grid level due to differences in spatial variation from different data sources. Furthermore we also adapted (annual and decadal, grid-level) information provided by the History Database of the Global Environment (HYDE version 3.2)^36,37^ to reconstruct the temporal trajectories of different land cover/uses (e.g., croplands, pastures, forest and other natural vegetated areas).

In the sections below, we first define the N surplus to agricultural and non-agricultural soils. Second, we provide details on the methodology for the reconstruction of the land use areas (cropland, pasture, forest, semi-natural vegetation, urban, non-vegetation) and crop specific harvested area (non-fodder and fodder crops). Third, we further explain the steps adopted to reconstruct the N inputs (through fertilizer, manure, atmospheric deposition and biological N fixation) and N outputs (N removal from cropland, pastures and forest).

### N surplus

We used the concept of N soil surface balance, which is the difference between N inputs and N output with adjustment of volatilization losses during manure storage system^11,20,26^. Here, total N surplus is the sum of N surplus over agriculture (cropland and pasture) and non-agriculture areas (forest, semi-natural vegetation, non-vegetation and urban) as given in Eq. (1) (all variables are in *kg ha*^−1^ *yr*^−1^):

1 $$Sur{p}_{{\rm{soil}}}(i,y)=Sur{p}_{{\rm{agri}}}(i,y)+Sur{p}_{{\rm{NonAgri}}}(i,y)$$

where *i* is grid cell; *y* is year; *Surp*_*soil*_ is the total N surplus; *Surp*_agri_ is the N surplus over agriculture area; and *Surp*_NonAgri_ is the N surplus over non-agriculture area. The individual component of N surplus are explained in the following sections.

#### N surplus over agriculture soils

N surplus over agricultural soils accounts for the N surplus over croplands (*Surp*_cr_) and pastures (*Surp*_past_). N surplus over croplands and pastures is estimated as the difference between the inputs to cropland and pasture (*Inp*_cr_ and *Inp*_past_, respectively) through mineral fertilizers (*FERT*_cr_ and *FERT*_past_, respectively), animal manure (*MAN*_cr_ and *MAN*_past_, respectively), biological N fixation (*BNF*_cr_ and *BNF*_past_, respectively) and atmospheric deposition (*DEP*_cr_ and *DEP*_past_, respectively), and the N outputs (*Out*_cr_ and *Out*_past_, respectively) through N removal via harvested crops (*Rem*_cr_) and via animal grazing and cutting of grass (*Rem*_past_), respectively. These can be summarized in Eqs. (2–8) (all variables are in *kg ha*^−1^ *yr*^−1^) as:

2 $$Sur{p}_{{\rm{agri}}}(i,y)=Sur{p}_{{\rm{cr}}}(i,y)+Sur{p}_{{\rm{past}}}(i,y)$$

3 $$Sur{p}_{{\rm{cr}}}(i,y)=In{p}_{{\rm{cr}}}(i,y)-Ou{t}_{{\rm{cr}}}(i,y)$$

4 $$In{p}_{{\rm{cr}}}(i,y)=FER{T}_{{\rm{cr}}}(i,y)+MA{N}_{{\rm{cr}}}(i,y)+DE{P}_{{\rm{cr}}}(i,y)+BN{F}_{{\rm{cr}}}(i,y)$$

5 $$Ou{t}_{{\rm{cr}}}(i,y)=Re{m}_{{\rm{cr}}}(i,y)$$

6 $$Sur{p}_{{\rm{past}}}(i,y)=In{p}_{{\rm{past}}}(i,y)-Ou{t}_{{\rm{past}}}(i,y)$$

7 $$In{p}_{{\rm{past}}}(i,y)=FER{T}_{{\rm{past}}}(i,y)+MA{N}_{{\rm{past}}}(i,y)+DE{P}_{{\rm{past}}}(i,y)+BN{F}_{{\rm{past}}}(i,y)$$

8 $$Ou{t}_{{\rm{past}}}(i,y)=Re{m}_{{\rm{past}}}(i,y)$$

#### N surplus over non-agriculture soils

N surplus over non-agriculture soils (*Surp*_NonAgri_) includes N surplus over forest (*Surp*_For_), semi-natural vegetation (*Surp*_NatVeg_), urban (*Surp*_Urban_) and rest non-vegetated areas (*Surp*_NonVeg_). N surplus over forest is derived as the difference between N inputs to forest (*Inp*_For_) through biological N fixation (*BNF*_For_) and atmospheric deposition (*DEP*_for_) and N outputs (*Out*_For_) via N removal from forest (*Rem*_For_). N surplus over semi-natural vegetation (*Surp*_NatVeg_) covers N inputs via atmospheric deposition (*DEP*_NatVeg_) and biological N fixation (*BNF*_NatVeg_). N surplus over the urban and rest non-vegetative areas (*Surp*_Urban_ and *Surp*_NonVeg_, respectively) is derived as N inputs via atmospheric deposition (*DEP*_Urban_ and *DEP*_NonVeg_, respectively). These can be summarized in Eqs. (9–15) (all variables are in *kg ha*^−1^ *yr*^−1^) as:

9 $$Sur{p}_{{\rm{NonAgri}}}(i,y)=Sur{p}_{{\rm{For}}}(i,y)+Sur{p}_{{\rm{NatVeg}}}(i,y)+Sur{p}_{{\rm{NonVeg}}}(i,y)+Sur{p}_{{\rm{Urban}}}(i,y)$$

10 $$Sur{p}_{{\rm{For}}}(i,y)=In{p}_{{\rm{For}}}(i,y)-Ou{t}_{{\rm{For}}}(i,y)$$

11 $$In{p}_{{\rm{For}}}(i,y)=DE{P}_{{\rm{For}}}(i,y)+BN{F}_{{\rm{For}}}(i,y)$$

12 $$Ou{t}_{{\rm{For}}}(i,y)=Re{m}_{{\rm{For}}}(i,y)$$

13 $$Sur{p}_{{\rm{NatVeg}}}(i,y)=DE{P}_{{\rm{NatVeg}}}(i,y)+BN{F}_{{\rm{NatVeg}}}(i,y)$$

14 $$Sur{p}_{{\rm{NonVeg}}}(i,y)=DE{P}_{{\rm{NonVeg}}}(i,y)$$

15 $$Sur{p}_{{\rm{Urban}}}(i,y)=DE{P}_{{\rm{Urban}}}(i,y)$$

### Land use categories

We compiled a range of databases to estimate annual trajectories of coverage areas for different land types including the harvested areas for 17 non-fodder crops and 6 fodder crop categories. These estimates were used in various steps to reconstruct the N surplus, for example to derive crop specific fertilizer application rates, distribution of mineral fertilizer and animal manure to cropland and pastures. In below, we provide details on the derivation of these areas from available databases.

#### Cropland, pasture and agriculture area

Cropland is defined as land used for cultivation of crops including arable crops and land under permanent crops^11,34,38^. Pasture area in our estimates is the land under permanent meadow and pasture^38^. First, to obtain the spatial variability at gridded level, we used the dataset from Ramankutty *et al*.^34^ which provides the global spatial distribution of cropland and pasture area (denoted as *A*_Ramankutty-crop_ (*ha*) and *A*_Ramankutty-past_ (*ha*), respectively) at a spatial resolution of 5 arcmin for the year around 2000. The land use data in Ramankutty *et al*.^34^ is derived based on agriculture inventories and land cover data from satellite imageries. To derive the temporal variability in the gridded values of cropland and pasture area, we adopted the land use dataset from the History Database of the Global Environment (HYDE version 3.2)^36^. HYDE provides global cropland and pasture area as decadal values for the period 1700–2000 and annual for the period 2000–2017 at a spatial resolution of 5 arcmin. We generated the annual time series of the cropland and pasture areas from HYDE (denoted as *A*_HYDE-cr_ (*ha*) and *A*_HYDE-past_ (*ha*), respectively) for the period 1850–2000 using linear interpolation for every two decadal estimates. For the year 2018 and 2019, we used the same value as of the year 2017. We derived the temporal variability of cropland and pasture area from the HYDE dataset for each year during 1850–2019, by referencing it to the year 2000 (*R*_HYDE-cr_ (-) and *R*_HYDE-past_ (-), respectively), as given in Eqs. (16) and (17):

16 $${R}_{{\rm{HYDE \mbox{-} cr}}\left(i,{y}_{1850\mbox{--}2019}\right)}=\frac{{A}_{{\rm{HYDE \mbox{-} cr}}}\left(i,{y}_{1850\mbox{--}2019}\right)}{{A}_{{\rm{HYDE \mbox{-} cr}}}\left(i,{y}_{2000}\right)}$$

17 $${R}_{{\rm{HYDE \mbox{-} past}}\left(i,{y}_{1850\mbox{--}2019}\right)}=\frac{{A}_{{\rm{HYDE \mbox{-} past}}}\left(i,{y}_{1850\mbox{--}2019}\right)}{{A}_{{\rm{HYDE \mbox{-} past}}}\left(i,{y}_{2000}\right)}$$

where *y*_2000_ is the year 2000; *y*_1850–2019_ is the year (in the period 1850–2019).

We then applied these normalised (temporal) values of cropland and pasture area of Eqs. (16–17) to the respective land use area of Ramankutty *et al*.^34^ in 2000, as given in Eqs. (18) and (19). By doing so, we maintained the spatial distribution of land use areas as of Ramankutty *et al*.^34^, while considering the annual temporal developments from HYDE. Finally, we derived agriculture area (*A*_agri_ (*ha*)) by combining our reconstructed gridded cropland and pasture area (*A*_cr_ (*ha*) and *A*_past_ (*ha*), respectively) for the time period 1850–2019, as given in Eq. (20).

18 $${A}_{{\rm{cr}}}\left(i,{y}_{1850{\rm{\mbox{--}}}2019}\right)={A}_{{\rm{Ramankutty \mbox{-} crop}}}\left(i,{y}_{2000}\right)\times {R}_{{\rm{HYDE \mbox{-} cr}}\left(i,{y}_{1850{\rm{\mbox{--}}}2019}\right)}$$

19 $${A}_{{\rm{past}}}\left(i,{y}_{1850\mbox{--}2019}\right)={A}_{{\rm{Ramankutty \mbox{-} past}}}\left(i,{y}_{2000}\right)\times {R}_{{\rm{HYDE \mbox{-} past}}\left(i,{y}_{1850\mbox{--}2019}\right)}$$

20 $${A}_{{\rm{agri}}}\left(i,{y}_{1850\mbox{--}2019}\right)={A}_{{\rm{cr}}}\left(i,{y}_{1850\mbox{--}2019}\right)+{A}_{{\rm{past}}}\left(i,{y}_{1850\mbox{--}2019}\right)$$

#### Harmonization of cropland, pasture and agriculture area at country-level with FAOSTAT

We used the FAOSTAT dataset as reference database for country-level information, due to its consistent availability for the period 1961–2019 and global coverage across a range of variables required to estimate the N surplus. To match our estimate of cropland and pasture area, we first derived “Cropland” and “Land and permanent meadows and pastures” from FAOSTAT for the time period 1961–2019. Then, for each year and country, we calculated country-level differences (as ratios) for cropland and pasture (${R}_{{A}_{{\rm{cr}}}}$ (-) and ${R}_{{A}_{{\rm{past}}}}$ (-), respectively) between those given in FAOSTAT (*A*_*FAO*-cr_ (*ha*) and *A*_*FAO*-past_ (*ha*), respectively) and ones estimated in our study (*A*_cr_ (*ha*) and *A*_past_ (*ha*)), as summarized in Eqs. (21–22):

21 $${R}_{{A}_{{\rm{cr}}}}(u,{y}_{1961\mbox{--}2019})=\frac{{A}_{FAO \mbox{-} {\rm{cr}}}\left(u,{y}_{1961\mbox{--}2019}\right)}{{\sum }_{i=1}^{{n}_{u}}\,{A}_{{\rm{cr}}}\left(i,{y}_{1961\mbox{--}2019}\right)}$$

22 $${R}_{{A}_{{\rm{past}}}}\left(u,{y}_{1961\mbox{--}2019}\right)=\frac{{A}_{FAO{\rm{ \mbox{-} past}}}\left(u,{y}_{1961\mbox{--}2019}\right)}{{\sum }_{i=1}^{{n}_{u}}{A}_{{\rm{past}}}\left(i,{y}_{1961\mbox{--}2019}\right)}$$

where *y*_1961_ is the year 1961; *y*_1961–2019_ is the year (in the period 1961–2019). *u* is country; *n*_*u*_ is the number of grid cell in the u-th country.

Then, we applied these calculated ratio to our gridded estimates of cropland and pasture areas to ensure that the FAOSTAT country totals are maintained for the period 1961–2019. The resulting gridded cropland and pasture areas (${C}_{{A}_{{\rm{cr}}}}(ha)$ and ${C}_{{A}_{{\rm{past}}}}(ha)$, respectively) can be given in Eqs. (23–24) as:

23 $${C}_{{A}_{{\rm{cr}}}}\left(i,{y}_{1961\mbox{--}2019}\right)={R}_{{A}_{{\rm{cr}}}}\left(u,{y}_{1961\mbox{--}2019}\right)\times {A}_{{\rm{cr}}}\left(i,{y}_{1961\mbox{--}2019}\right)$$

24 $${C}_{{A}_{{\rm{past}}}}\left(i,{y}_{1961\mbox{--}2019}\right)={R}_{{A}_{{\rm{past}}}}\left(u,{y}_{1961\mbox{--}2019}\right)\times {A}_{{\rm{past}}}\left(i,{y}_{1961\mbox{--}2019}\right)$$

As FAOSTAT does not provide estimates before 1961, we applied the same ratio as of 1961 for the time period 1850–1960 as given in Eqs. (25–26):

25 $${C}_{{A}_{{\rm{cr}}}}\left(i,{y}_{1850\mbox{--}1960}\right)={R}_{{A}_{{\rm{cr}}}}\left(u,{y}_{1961}\right)\times {A}_{{\rm{cr}}}\left(i,{y}_{1850\mbox{--}1960}\right)$$

26 $${C}_{{A}_{{\rm{past}}}}\left(i,{y}_{1850\mbox{--}1960}\right)={R}_{{A}_{{\rm{past}}}}\left(u,{y}_{1961}\right)\times {A}_{{\rm{past}}}\left(i,{y}_{1850\mbox{--}1960}\right)$$

For the countries for which FAOSTAT data are missing before 1992, such as Croatia, Estonia, Latvia, Lithuania and Slovenia, we applied the same ratio as of the year 1992 for the period 1850–1991. Furthermore, for the countries like Belgium and Luxembourg, Czech Republic and Slovakia, the FAOSTAT maintains single (combined) values of reported variables for the past records (prior to 1993 for Czechoslovakia and before 2000 for Belgium-Luxembourg). In our estimation of country-specific ratios we took care of these details, and accordingly applied a single ratio factor for the adjoining countries and records. Finally, the agricultural area was derived as a sum of the FAOTSTAT harmonized cropland and pasture area as mentioned in Eq. (27):

27 $${C}_{{A}_{{\rm{agri}}}}\left(i,{y}_{1850\mbox{--}2019}\right)={C}_{{A}_{{\rm{cr}}}}\left(i,{y}_{1850\mbox{--}2019}\right)+{C}_{{A}_{{\rm{past}}}}\left(i,{y}_{1850\mbox{--}2019}\right)$$

where *y*_1961_ is the year 1961; *y*_1850–1960_ is the year (in the period 1850–1960).

Finally, we checked for a physical consistency of grid-level reconstructed area estimates i.e., the exceedance of the gridded agriculture area of Eq. (27) (${C}_{{A}_{{\rm{agri}}}}(ha)$) beyond the physical area of a given grid cell. This condition, although appeared seldom, was unavoidable due to differences in origin of different databases (e.g., FAOSTAT, HYDE, Ramankutty *et al*.^34^) used in our reconstruction. To maintain the physical consistency, we equally redistributed the agricultural area in excess to other grid cells in a way that the agricultural area in a grid cell is not greater than the area of a grid cell. In a similar way, we corrected the gridded cropland areas (${C}_{{A}_{{\rm{cr}}}}(ha)$) and pasture areas (${C}_{{A}_{{\rm{past}}}}(ha)$) to guarantee that FAOSTAT country totals are satisfied and that the sum of cropland area and pasture area does not exceed the total agricultural area in a given grid cell.

#### Reconstruction of non-agriculture area

We derived the non-agricultural area in a given grid cell as the area *A*_NonAgri_ (*ha*) that is left after allocation of cropland and pasture area, as given in Eq. (28):

28 $${A}_{{\rm{NonAgri}}}\left(i,{y}_{1850\mbox{--}2019}\right)=1-\left[{C}_{{A}_{{\rm{cr}}}}\left(i,{y}_{1850\mbox{--}2019}\right)+{C}_{{A}_{{\rm{past}}}}\left(i,{y}_{1850\mbox{--}2019}\right)\right]$$

Afterwards, we utilized the classification of land cover categories from global land cover (GLC)^39^ that is available at a spatial resolution of 300 m and for the year 2000. GLC includes 23 land cover classes that we grouped into 5 categories namely, cropland, semi-natural-vegetation (tree, shrub-land, herbaceous cover), forest (broad-leaved, evergreen and deciduous forest), non-vegetation (bare areas, water bodies) and urban area. We calculated the fractions of forest, semi-natural-vegetation, non-vegetation and urban area (*For*_*GLC*_, *NatVeg*_*GLC*_, *NonVeg*_*GLC*_, *Urban*_*GLC*_, (-) respectively) from GLC land cover within our target grid cell of 5 arcmin, and their sum which corresponds to the total non-agricultural fraction (*NonAgri*_*GLC*_ (-)). We then estimated the relative proportion of forest, semi-natural vegetation, non-vegetation and urban area $f{r}_{Fo{r}_{GLC}}$, $f{r}_{NatVe{g}_{GLC}}$, $f{r}_{NonVe{g}_{GLC}}$, $f{r}_{Urba{n}_{GLC}}$, (-) respectively) within the total non-agricultural fraction as given from Eqs. (29) to (32):

29 $$f{r}_{Fo{r}_{GLC}}\left(i,{y}_{2000}\right)=\frac{Fo{r}_{GLC}\left(i,{y}_{2000}\right)}{NonAgr{i}_{GLC}\left(i,{y}_{2000}\right)}$$

30 $$f{r}_{NatVe{g}_{GLC}}\left(i,{y}_{2000}\right)=\frac{NatVe{g}_{GLC}\left(i,{y}_{2000}\right)}{NonAgr{i}_{GLC}\left(i,{y}_{2000}\right)}$$

31 $$f{r}_{Urba{n}_{GLC}}\left(i,{y}_{2000}\right)=\frac{Urba{n}_{GLC}\left(i,{y}_{2000}\right)}{NonAgr{i}_{GLC}\left(i,{y}_{2000}\right)}$$

32 $$f{r}_{NonVe{g}_{GLC}}\left(i,{y}_{2000}\right)=\frac{NonVe{g}_{GLC}\left(i,{y}_{2000}\right)}{NonAgr{i}_{GLC}\left(i,{y}_{2000}\right)}$$

To infer the annual development of forest, semi-natural vegetation, non-vegetation and urban land cover areas (*A*_*For*_, *A*_*NatVeg*_, *A*_*NonVeg*_ and *A*_*Urban*_ (*ha*), respectively), we multiplied the corresponding relative fractions (Eqs. (29) to (32)) with the gridded non-agricultural area (*A*_*NonAgri*_ (*ha*)) of Eq. (28), as summarised in Eqs. (33) to (36):

33 $${A}_{For}\left(i,{y}_{1850\mbox{--}2019}\right)=f{r}_{For}\left(i,{y}_{2000}\right)\times {A}_{NonAgri}\left(i,{y}_{1850\mbox{--}2019}\right)$$

34 $${A}_{NatVeg}\left(i,{y}_{1850\mbox{--}2019}\right)=f{r}_{NatVeg}\left(i,{y}_{2000}\right)\times {A}_{NonAgri}\left(i,{y}_{1850\mbox{--}2019}\right)$$

35 $${A}_{NonVeg}\left(i,{y}_{1850\mbox{--}2019}\right)=f{r}_{NonVeg}\left(i,{y}_{2000}\right)\times {A}_{NonAgri}\left(i,{y}_{1850\mbox{--}2019}\right)$$

36 $${A}_{Urban}\left(i,{y}_{1850\mbox{--}2019}\right)=f{r}_{Urban}\left(i,{y}_{2000}\right)\times {A}_{NonAgri}\left(i,{y}_{1850\mbox{--}2019}\right)$$

### Crop specific harvested area

#### Non-fodder crops

We obtained gridded crop specific harvested areas from Monfreda *et al*.^35^ which is available for 175 different crop types and that correspond to the representative year of 2000. Monfreda *et al*.^35^ generated crop harvested area by combining information from surveys and agricultural census for around 206 countries. In addition to national statistics, a substantial information in these databases is derived from Agro-MAPS which is a project of combined efforts from FAOSTAT, the International Food Policy Research Institute (IFPRI), and the Center for Sustainability and the Global Environment (SAGE)^35^. Among these 175 crops, we selected 17 major crops for which fertilizer application rates are provided^40^ and that have either a large production across Europe or a N content greater than 1 *kg* of N tonne^−1^ of product. The selected crops are listed in Table 2.

**Table 2:** N content and biological N fixation rates for crops and other land types included in this study.

Crop/Land type	N content	BNF rate
	*kg* of N *t*^−1^ of product	*kg* of N *ha*^−1^ of area
**Non-fodder crops**		
Wheat	20^8,18^	—
Maize	13^8,18^	—
Rice	12^8,24^	—
Soybean	45^8,18^	80^18,68^
Sunflower seed	23^18,24^	—
Millet	24^18,24^	—
Sorghum	14^24^	—
Rye	22^18,24^	—
Barley	18^18,24^	—
Oats	20^18,24^	—
Triticale, Buckwheat	16^24^	—
Rapeseed, Sesame	35^24^	—
Pulses	40^8,18^	50^8,18^
Sugar-beet	1.5^18,24^	—
Potato	3^18,24^	—
**Fodder crops**		
Temporary grassland, lucerne,green maize, root crops etc.	—^21^	—^21^
**Forest**		
Temperate	—	16^54^
Boreal	—	1.77^54^
**Pasture**	—	5^8^
**Semi-natural vegetation**	—	2.7^54^

The values for N content and BNF rate were derived based on estimates reported in previous studies^8,18,24,68^.

To construct annual estimates of crop specific harvested area (*A*_crops_ (*ha*)), we first applied temporal dynamics of cropland area (${C}_{{A}_{{\rm{cr}}}}(ha)$) derived in Eqs. (23) and (25) with respect to the gridded dataset from Monfreda *et al*.^35^ (${A}_{{{\rm{crops}}}_{Monfreda}}(ha)$); as given in Eq. (37). Consequently, the temporal dynamics in our estimates are referenced to the year 2000 to maintain the consistency with the Monfreda *et al*.^35^ database and the overall methodology applied in the reconstruction of cropland/agricultural areas (as described above).

37 $${A}_{{\rm{crops}}}\left(i,c,{y}_{1850\mbox{--}2019}\right)={A}_{{{\rm{crops}}}_{Monfreda}}\left(i,c,{y}_{2000}\right)\times \frac{{C}_{{A}_{{\rm{cr}}}}\left(i,{y}_{1850\mbox{--}2019}\right)}{{C}_{{A}_{{\rm{cr}}}}\left(i,{y}_{2000}\right)}$$

where *c* is crop, *y*_1850–2019_ is the year (in the period 1850–2019).

We then ensured that the calculated crop specific harvested areas match estimates from FAOSTAT at a country level. To do so, we derived the country level ratio (*R*_*A*_ (-)) between crop harvested area from FAOSTAT (${A}_{crop{s}_{FAO}}(ha)$) and crop area provided in this study, as given in Eq. (38):

38 $${R}_{A}\left(u,c,{y}_{1961\mbox{--}2019}\right)=\frac{{A}_{crop{s}_{FAO}}\left(u,c,{y}_{1961\mbox{--}2019}\right)}{{\sum }_{i=1}^{{n}_{u}}\,{A}_{{\rm{crops}}}\left(u,c,{y}_{1961\mbox{--}2019}\right)}$$

The calculated ratio was then applied to the estimates of gridded annual crop specific harvested area (*A*_crops_ (*ha*)) to obtain the crop harvested area harmonized at country level with FAOSTAT (${C}_{{A}_{crops}}(ha)$), as mentioned in Eq. (39). Since FAOSTAT data only starts in 1961, all years prior to 1961 have been rescaled by the scaling factor (ratio) of 1961 as given in Eq. (40):

39 $${C}_{{A}_{crops}}\left(i,c,{y}_{1961\mbox{--}2019}\right)={A}_{{\rm{crops}}}\left(i,c,{y}_{1961\mbox{--}2019}\right)\times {R}_{A}\left(u,c,{y}_{1961\mbox{--}2019}\right)$$

40 $${C}_{{A}_{crops}}\left(i,c,{y}_{1850\mbox{--}1960}\right)={A}_{{\rm{crops}}}\left(i,c,{y}_{1850\mbox{--}1960}\right)\times {R}_{A}\left(u,c,{y}_{1961}\right)$$

#### Fodder crops

Since FAOSTAT does not provide data on fodder crops, we used for this the crop areas from Einarsson *et al*.^21^. This dataset is available for 26 European countries during 1961–2019. The dataset is given for 6 fodder crop categories (Table 2) namely: temporary grassland, lucerne, other leguminous plants, green maize, plants harvested from arable land and other root crops (forage beet, turnip etc.). For the temporal development of fodder crop area during 1850–1960, we utilized the temporal variability of the reconstructed cropland area (see Eqs. (23) and (25)). To do so, we first derived country-level ratio (${R}_{{A}_{fodder}}$(-)) of fodder crop area (*A*_*fodder*_ (*ha*)) to the cropland area (${C}_{{A}_{{\rm{cr}}}}(ha)$) in the year 1961 as given in Eq. (41). Later, we applied the country-level ratio of the year 1960, to the cropland year during 1850–1960, as given in Eq. (42).

41 $${R}_{{A}_{fodder}}\left(u,c,{y}_{1961}\right)=\frac{{A}_{fodder}\left(u,c,{y}_{1961}\right)}{{\sum }_{i=1}^{{n}_{u}}{C}_{{A}_{{\rm{cr}}}}\left(i,{y}_{1961}\right)}$$

42 $${A}_{fodder}\left(u,c,{y}_{1850\mbox{--}1960}\right)={R}_{{A}_{fodder}}\left(u,c,{y}_{1961}\right)\times \mathop{\sum }\limits_{i=1}^{{n}_{u}}{C}_{{A}_{{\rm{cr}}}}\left(i,{y}_{1850\mbox{--}1960}\right)$$

where *c* is fodder crop, *y*_1961_ is the year 1961, *y*_1850–1960_ is the year (in the period 1850–1960).

Einarsson *et al*.^21^ does not provide fodder crop area before 1992 for some countries such as Croatia, Estonia, Latvia, Lithuania, Slovenia, Czechia, Slovakia, Belgium and Luxembourg. To fill the data gaps in fodder crop area, following consideration were made:

- Where possible, we extrapolated country level ratio (${R}_{{A}_{fodder}}$(-)) from neighbouring countries with similar climate and geographical conditions. For instance, the country level ratio (${R}_{{A}_{fodder}}$(-)) of Austria, Sweden, Romania, Greece was used for Switzerland, Norway, Ukraine, Turkey, respectively.
- For the other missing countries that are located in Eastern Europe (e.g. Croatia, Estonia), we used an aggregated country-level ratio (${R}_{{A}_{fodder}}$(-)) from other available countries in Eastern Europe namely Bulgaria, Romania, Hungary for the year 1992. This ratio was then applied to the cropland area of the missing countries during 1850–1991.

We thus completed the time series of fodder crop area for the period 1850–2019 at country-level, that we downscaled to gridded level using the fodder specific crop area from Monfreda *et al*.^35^, similar to the approach described above for the reconstruction of non-fodder crop areas.

### N inputs

N inputs in our estimates consisted of mineral fertilizer, animal manure, atmospheric deposition and biological fixation to agriculture and non-agriculture soil at gridded level from 1850 to 2019.

#### Fertilizer

The amount of fertilizers applied to croplands and pastures is generally assessed using application rates for the different crop types and pastures^1,30^. In particular, a recent study suggests that a large share of fertilizers are applied to permanent pastures in many European countries^21^. However, differences in the fractions of fertilizer applied to croplands and pastures have been reported as being a major source of uncertainty^5^. To account for these uncertainties, we derived two gridded estimates of fertilizer application based on two different sets of application rates for croplands and pastures. These two sets of rates were then used to downscale the country-level fertilizer amounts applied to soil at a gridded level.

#### Country-level fertilizer applied to soil during 1850–2019

During 1961 to 2019, we derived country-level data of fertilizer applied to agricultural soils from FAOSTAT^41^. For a few countries, namely Croatia, Estonia, Latvia, Lithuania, Belarus, Ukraine, no data are provided before 1992. For these countries, we estimated the N fertilizer application ($N\;fe{r}_{Missin{g}_{Eas{t}_{EU}}}$ (*kg yr*^−1^)) during the period 1961–1991 by applying the temporal dynamics of Eastern European countries that have reported data i.e., Bulgaria, Hungary and Czechoslovakia, as given in Eq. (43):

43 $$N\;fe{r}_{Missin{g}_{Eas{t}_{EU}}}(u,{y}_{1961\mbox{--}1991})=\frac{N\;fe{r}_{Eas{t}_{EU}}({y}_{1961\mbox{--}1991})}{N\;fe{r}_{Eas{t}_{EU}}({y}_{1992})}\times N\;fe{r}_{Missin{g}_{Eas{t}_{EU}}}(u,{y}_{1992})$$

where $N\;fe{r}_{Eas{t}_{EU}}$ (*kg yr*^−1^) is the total fertilizer application calculated over Bulgaria, Hungary and Czechoslovakia. Moreover, Belgium and Luxembourg are reported in FAOSTAT as a single country during 1961–1999, while separate country estimates are provided between 2000–2019. Similarly, Czechia and Slovakia is reported in a single database of Czechoslovakia between 1961–1992. Before 2000 for Belgium and Luxembourg and before 1993 for Czechia and Slovakia, we applied the dynamics of Belgium-Luxembourg and former Czechoslovakia, respectively.

During 1850–1960, we lacked country-level information from FAOSTAT. During 1850–1920, we assumed that there is no fertilizer applied to agricultural soil based on evidence from previous studies^1,42^. Regarding the time period of 1920–1960, we utilized the temporal dynamics from Holland *et al*.^42^ that provides global estimates of fertilizer production during 1925–1960 based on different data sources^43,44^. We filled the values of global fertilizer production for the years between 1920 and 1924 using linear interpolation with no (or zero) fertilizer in the year 1920 and Holland *et al*.^42^ estimates for the year 1925. The global temporal dynamic was then applied to all countries in the study domain between 1920–1960 with respect to their corresponding estimates for the year 1961. We termed the completed annual country-level fertilizer amount as $N\;fe{r}_{soil}(u,{y}_{1850\mbox{--}2019})$.

#### Distribution of fertilizer to croplands and pastures

To downscale the annual country-level fertilizer amount to croplands and pastures at grid level, we utilized two different sets of application rates to account for the uncertainties in the gridded spatial pattern of fertilizer application within a given country. We first derived country-level fertilizer application rates for individual crops and grassland based on information given by the International Fertilizer Industry Association (IFA; https://www.ifastat.org), which are then adjusted using two different approaches. We provide further details in the paragraphs below.

We obtained country-level fertilizer use per crop types and grassland (*N fer*_*crops*_ and *N fer*_*grass*_, respectively (*kg yr*^−1^) from IFA in 2014–2015^40^. IFA provides country-level total amount of N fertilizer use for 13 crop groups, i.e., wheat, rice, maize (both grain and silage), other cereals (barley, sorghum, oats, rye, triticale, millet, etc.), soybean, oil palm, other oil seeds (rapeseed, mustard, sunflower, groundnut, coconut, etc), fiber crops (cotton, flax, hemp, jute, etc), sugar crops (sugar cane and sugar beet), roots and tubers, fruits, vegetables, grassland (pastures for hay, silage and grazing, and temporary grasslands) and residual (pulses, nut trees, rubber, cocoa, coffee, tea, tobacco, etc.). In Europe, IFA data does not contain the N fertilizer use for each individual country but it reports the total value for EU-28, as well as values for Ukraine, Russia and Belarus. We combined fertilizer use amount from IFA and harvested areas of crops and grassland from FAOSTAT^38^ (*A*_*crops*_ and *A*_*grass*_ (*ha*), respectively) to estimate the respective application rates. The area of grassland includes temporary grasslands (“land under temporary meadows and pastures” in FAOSTAT) and pastures (“land under permanent meadows and pastures” in FAOSTAT). We thus derived country-level fertilizer application rates for individual non-fodder crops ($N\;fe{r}_{crop{s}_{Rate}}$ (*kg ha*^−1^) of crop harvested areas *yr*^−1^) and grassland ($N\;fe{r}_{gras{s}_{Rate}}$ (*kg ha*^−1^) of grassland areas *yr*^−1^), as summarized in Eqs. (44–45):

44 $$N\;fe{r}_{crop{s}_{Rate}}(u,c,{y}_{2015})=\frac{N\;fe{r}_{crops}(u,c,{y}_{2015})}{{A}_{crops}(u,c,{y}_{2015})}$$

45 $$N\;fe{r}_{gras{s}_{Rate}}(u,{y}_{2015})=\frac{N\;fe{r}_{grass}(u,{y}_{2015})}{{A}_{grass}(u,{y}_{2015})}$$

For fodder crops and pastures, we adopted the fertilizer application rates for the grassland (Eq. (45)). For the rest of the countries in our domain that are not the part of the IFA dataset, we assumed the same fertilizer application rates for individual crops and pastures as of EU-28.

Afterwards, to inform the spatial variability, we multiplied the country-level fertilizer application rates for non-fodder crops ($N\;fe{r}_{crop{s}_{Rate}}$) and grassland ($N\;fe{r}_{gras{s}_{Rate}}$) by the gridded areas of non-fodder crops, pastures and fodder crops (${C}_{{A}_{{\rm{crops}}}}$, ${C}_{{A}_{{\rm{past}}}}$ and *A*_fodder_ respectively, (*ha*)) during 1850–2019. Consequently, we obtained annual fertilizer application amount for each crop type (non-fodder and fodder), pastures, and total (*N fer*_crops_, *N fer*_fodder_, *N fer*_past_, *N fer*_soil_, respectively (*kg yr*^−1^)) for each grid cell, as summarised in Eqs. (46–49):

46 $$N\;fe{r}_{{\rm{crops}}}\left(i,c,{y}_{1850\mbox{--}2019}\right)=N\;fe{r}_{crop{s}_{Rate}}\left(u,c,{y}_{2015}\right)\times {C}_{{A}_{{\rm{crops}}}}\left(i,c,{y}_{1850\mbox{--}2019}\right)$$

47 $$N\;fe{r}_{{\rm{past}}}\left(i,{y}_{1850\mbox{--}2019}\right)=N\;fe{r}_{gras{s}_{Rate}}\left(u,{y}_{2015}\right)\times {C}_{{A}_{{\rm{past}}}}\left(i,{y}_{1850\mbox{--}2019}\right)$$

48 $$N\;fe{r}_{{\rm{fodder}}}\left(i,c,{y}_{1850{\rm{\mbox{--}}}2019}\right)=N\;fe{r}_{gras{s}_{Rate}}\left(u,{y}_{2015}\right)\times {A}_{fodder}\left(i,c,{y}_{1850{\rm{\mbox{--}}}2019}\right)$$

49 $$N\;fe{r}_{{\rm{soil}}}\left(i,{y}_{1850{\rm{\mbox{--}}}2019}\right)=\mathop{\sum }\limits_{i=1}^{{n}_{c}}N\;fe{r}_{{\rm{crops}}}\left(i,c,{y}_{1850{\rm{\mbox{--}}}2019}\right)+N\;fe{r}_{{\rm{past}}}\left(i,{y}_{1850{\rm{\mbox{--}}}2019}\right)+\mathop{\sum }\limits_{i=1}^{{n}_{c}}N\;fe{r}_{{\rm{fodder}}}\left(i,c,{y}_{1850{\rm{\mbox{--}}}2019}\right)$$

In the next step, we adjusted the fertilizer application amount (calculated in Eq. (49)) to ensure that it is consistent with the country-level fertilizer applied to soil during 1850–2019 reconstructed in previous steps (*Nfer*_soil_ (*kg yr*^−1^)). For this purpose, we first derived an adjustment factor (as ratio) of the country-level fertilizer amount to gridded fertilizer amount aggregated to country level. The calculated ratio was then multiplied by the fertilizer application rates of individual crops and grassland ($N\;fe{r}_{crop{s}_{Rate}}$ (*kg ha*^−1^) of crop harvested areas *yr*^−1^) and $N\;fe{r}_{gras{s}_{Rate}}$ (*kg ha*^−1^) of grassland areas *yr*^−1^), respectively) to derive adjusted fertilizer application rates for crops and grassland (${C}_{Nfe{r}_{crop{s}_{Rate}}}$ (*kg ha*^−1^) of crop harvested areas *yr*^−1^) and ${C}_{Nfe{r}_{gras{s}_{Rate}}}$ (*kg ha*^−1^) of grassland areas *yr*^−1^), respectively), as given from Eqs. (50–51):

50 $${C}_{Nfe{r}_{crop{s}_{Rate}}}\left(u,c,{y}_{1850\mbox{--}2019}\right)=N\;fe{r}_{crop{s}_{Rate}}\left(u,c,{y}_{2015}\right)\times \frac{N\;fe{r}_{soil}\left(u,{y}_{1850\mbox{--}2019}\right)}{{\sum }_{i=1}^{{n}_{u}}\,N\,fe{r}_{soil}\left(i,{y}_{1850\mbox{--}2019}\right)}$$

51 $${C}_{Nfe{r}_{gras{s}_{Rate}}}\left(u,{y}_{1850\mbox{--}2019}\right)=N\;fe{r}_{gras{s}_{Rate}}\left(u,{y}_{2015}\right)\times \frac{N\;fe{r}_{soil}\left(u,{y}_{1850\mbox{--}2019}\right)}{{\sum }_{i=1}^{{n}_{u}}\;N\,fe{r}_{soil}\left(i,{y}_{1850\mbox{--}2019}\right)}$$

Now to determine the partitioning of fertilizer application amount to croplands and pastures, we considered two different sets of application rates for croplands and pastures to account for (methodological) uncertainties. In the first approach, we adopted the application rates based on IFA rates along with their subsequent adjustments as detailed above in Eqs. (50) and (51). In the second approach, we further adjusted these rates so that the proportions of fertilizer applied to croplands and pastures are consistent with the (partitioning) information provided by Einarsson *et al*.^21^ for the period 1961–2019. For the countries with incomplete record in Einarsson *et al*.^21^, we used the proportions from either neighbouring countries or the average value of countries in Eastern Europe to fill the missing years, similar to the procedure we adopted in section “Crop specific harvested area-Fodder crops”.

Finally, from the two sets of fertilizer application rates, we estimated the gridded fertilizer amount applied to croplands and pastures (*N fer*_cr_ (*kg yr*^−1^) and *N fer*_*past*_ (*kg yr*^−1^), respectively) using the gridded areas of non-fodder crops, fodder crops and pastures. For instance, for the first approach (using the application rates of Eqs. (50) and (51)), the equations can be summarized as:

52 $$\begin{array}{ll}N\;fe{r}_{cr}\left(i,{y}_{1850\mbox{--}2019}\right) & =\mathop{\sum }\limits_{i=1}^{{n}_{c}}\left({C}_{Nfe{r}_{crop{s}_{Rate}}}\left(u,c,{y}_{1850\mbox{--}2019}\right)\times {C}_{{A}_{{\rm{crops}}}}\left(i,c,{y}_{1850\mbox{--}2019}\right)\right)\\  & \,+\mathop{\sum }\limits_{i=1}^{{n}_{c}}\left({C}_{Nfe{r}_{gras{s}_{Rate}}}\left(u,{y}_{1850\mbox{--}2019}\right)\times {A}_{fodder}\left(i,c,{y}_{1850\mbox{--}2019}\right)\right)\end{array}$$

53 $$N\;fe{r}_{past}\left(i,{y}_{1850\mbox{--}2019}\right)={C}_{Nfe{r}_{gras{s}_{Rate}}}\left(u,{y}_{1850\mbox{--}2019}\right)\times {C}_{{A}_{{\rm{past}}}}\left(i,{y}_{1850\mbox{--}2019}\right)$$

For the second approach, we replaced the fertilizer application rates that are used in Eqs. (52) and (53) (${C}_{Nfe{r}_{crop{s}_{Rate}}}$ and ${C}_{Nfe{r}_{gras{s}_{Rate}}}$) by the adjusted rates based on information provided by Einarsson *et al*.^21^.

Within each approach, the total gridded fertilizer amount applied to soil was then derived by summing the fertilizer applied to croplands and pastures. We thus obtained two different datasets of fertilizer application that reflect the methodological uncertainties in the gridded spatial pattern within a given country, while the country-level values are the same in the both datasets.

#### Animal manure

The amount of N excreted by the livestock (also called manure production) is typically calculated using N excretion rates and livestock counts. In addition, it can have different fates i.e., it can be left on pasture or it can be collected, stored and then applied to soils (cropland and pasture). The differences in livestock N excretion amounts obtained using different N excretion rates and the distribution of manure production between cropland and pasture has been recognized as another major source of uncertainty in N budget estimations^5,27,28^.

To account for these uncertainties, we utilized information from two datasets of animal manure (i.e., FAOSTAT^45^ and Einarsson *et al*.^21^) available at country-level during 1961–2019. Both datasets differ in estimating the total N excretion from livestock category due to different data input sources and methodology. For example, FAOSTAT calculates the total amount of N excreted by the livestock categories by multiplying the country-level livestock counts by regional-level values of typical animal mass and N excretion rates from the Intergovernmental Panel on Climate Change (IPCC)^46^. Einarsson *et al*.^21^, on the other hand, follows the approach of Lassaletta *et al*.^47^ in which the N excretion rates within a given livestock category are assumed to be proportional to their slaughter weight. Hence the N excretion rates are derived by multiplying the slaughtered animal weights by the ratio of N excretion rate to slaughtered animal weight for each country and year. The derived N excretion rates are then multiplied by the livestock counts to calculate total N excretion in each country and year. We included both these databases in our analysis. Note that for the missing estimates (for countries and time-periods) in the database from Einarsson *et al*.^21^, we adapted the same methodology as explained in section “Crop specific harvested area-Fodder crops”. We derived country-level data of manure applied to soils ($Ma{n}_{ap{p}_{soil}}$ (*kg yr*^−1^)) and manure left on pastures ($Ma{n}_{lef{t}_{past}}$ (*kg yr*^−1^)) for the period 1961–2019 from FAOSTAT^48^ and Einarsson *et al*.^21^. As we focus on the soil N-budget, the manure applied to soils in both databases excludes losses of reduced forms of N via volatilization occurring during manure management and storage. Subsequent deposition of these reduced forms of N is accounted for in the budget in the estimates of atmospheric N deposition.

To downscale the country-level values to gridded level, we utilized gridded information of manure production from Zhang *et al*.^29^ that was derived using the spatial distribution of livestock counts from the Global Livestock Impact Mapping System (GLIMS)^49^ and N excretion coefficients from the IPCC at a 5 arcmin spatial resolution for the time period 1860–2014. Zhang *et al*.^29^ does not give estimate of gridded manure production before 1860 and after 2015. We assumed the same amount of manure production for the time period 1850–1859 as of the year 1860 and for the time period 2015–2019 as of the year 2014. We combined the gridded manure production (*Man*_*prod*_ (*kg ha*^−1^) of grid area *yr*^−1^)) of Zhang *et al*.^29^ and the grid cell area (*A*_*grid*_ (*ha*)) to calculate the country-level ratio of manure applied to soil to manure production (${R}_{ap{p}_{soil}/prod}$ (-)) and manure left on pastures to manure production (${R}_{lef{t}_{past}/prod}$ (-)), given below in the Eqs. (54–55). Note that these ratios were estimated for each of two manure databases^21,45^, separately.

54 $${R}_{ap{p}_{soil}/prod}\left(u,{y}_{1961\mbox{--}2019}\right)=\frac{Ma{n}_{ap{p}_{soil}}\left(u,{y}_{1961\mbox{--}2019}\right)}{{\sum }_{i=1}^{{n}_{u}}\,Ma{n}_{prod}\left(i,{y}_{1961\mbox{--}2019}\right)\times {A}_{grid}(i)}$$

55 $${R}_{lef{t}_{past}/prod}\left(u,{y}_{1961\mbox{--}2019}\right)=\frac{Ma{n}_{lef{t}_{past}}\left(u,{y}_{1961\mbox{--}2019}\right)}{{\sum }_{i=1}^{{n}_{u}}\,Ma{n}_{prod}\left(i,{y}_{1961\mbox{--}2019}\right)\times {A}_{grid}(i)}$$

Next, we estimated the gridded rates of manure applied to soil and left on pastures ($Ma{n}_{ap{p}_{soil}}$ and $Ma{n}_{lef{t}_{past}}$, respectively, both are given in *kg ha*^−1^ of grid area *yr*^−1^) by multiplying the calculated ratios from Eqs. (54) and (55) by the gridded manure production from Zhang *et al*.^29^ (*Man*_*prod*_ (*kg ha*^−1^) of grid area *yr*^−1^)), as presented in Eqs. (56–57).

56 $$Ma{n}_{ap{p}_{soil}}\left(i,{y}_{1961\mbox{--}2019}\right)={R}_{ap{p}_{soil}/prod}\left(u,{y}_{1961\mbox{--}2019}\right)\times Ma{n}_{prod}\left(i,{y}_{1961\mbox{--}2019}\right)$$

57 $$Ma{n}_{lef{t}_{past}}\left(i,{y}_{1961\mbox{--}2019}\right)={R}_{lef{t}_{past}/prod}\left(u,{y}_{1961\mbox{--}2019}\right)\times Ma{n}_{prod}\left(i,{y}_{1961\mbox{--}2019}\right)$$

For the time period during 1850–1960, the same ratio of the year 1961 was applied to the manure production database, as given in Eqs. (58) and (59).

58 $$Ma{n}_{ap{p}_{soil}}\left(i,{y}_{1850\mbox{--}1960}\right)={R}_{ap{p}_{soil}/prod}\left(u,{y}_{1961}\right)\times Ma{n}_{prod}\left(i,{y}_{1850\mbox{--}1960}\right)$$

59 $$Ma{n}_{lef{t}_{past}}\left(i,{y}_{1850\mbox{--}1960}\right)={R}_{lef{t}_{past}/prod}\left(u,{y}_{1961}\right)\times Ma{n}_{prod}\left(i,{y}_{1850\mbox{--}1960}\right)$$

Finally, we checked the consistency of gridded manure application rates to fall within a maximum permissible limit following Bouwman *et al*.^50^ for European countries (170–250 *kg* *ha*^−1^ *of agricultural area yr*^−1^). In grid cells and years, where this maximum limit was crossed, the extra manure was redistributed to the neighbouring agricultural cells.

#### Distribution of manure applied between cropland and pasture

To distribute manure applied to soil (calculated in Eqs. (56) and (58) from both FAOSTAT and Einarsson *et al*.^21^ data) between cropland and pasture, we used two different methodologies to account for uncertainties. First, following a similar approach as Tian *et al*.^51^, we assumed the uniform manure application rates between cropland and pasture within a given grid cell. Thus, manure applied to cropland (*Man*_*cr*_ (*kg yr*^−1^)) was manure applied to soil (*Man*_*app/soil*_ (*kg ha*^−1^ *yr*^−1^)) times cropland area (${C}_{{A}_{{\rm{cr}}}}$ (*ha*)) in a given grid cell according to Eq. (60). Similarly, manure applied to pastures ($Ma{n}_{ap{p}_{past}}$ (*kg yr*^−1^)) was manure applied to soil (*Man*_*app/soil*_ (*kg ha*^−1^ *yr*^−1^)) times pasture area (${C}_{{A}_{{\rm{past}}}}$ (*ha*)) in a given grid cell, as given in Eq. (61). The final manure amount to pastures (*Man*_*past*_ (*kg yr*^−1^)) was derived as a sum of manure applied and left on pasture as expressed in Eq. (62).

60 $$Ma{n}_{cr}\left(i,{y}_{1850\mbox{--}2019}\right)=Ma{n}_{app/soil}\left(i,{y}_{1850\mbox{--}2019}\right)\times {C}_{{A}_{{\rm{cr}}}}\left(i,{y}_{1850\mbox{--}1960}\right)$$

61 $$Ma{n}_{ap{p}_{past}}(i,{y}_{1850\mbox{--}2019})=Ma{n}_{app/soil}(i,{y}_{1850\mbox{--}2019})\times {C}_{{A}_{{\rm{past}}}}(i,{y}_{1850\mbox{--}1960})$$

62 $$Ma{n}_{past}\left(i,{y}_{1850\mbox{--}2019}\right)=Ma{n}_{ap{p}_{past}}\left(i,{y}_{1850\mbox{--}2019}\right)+Ma{n}_{lef{t}_{past}}\left(i,{y}_{1850\mbox{--}1960}\right)$$

In the second manure application approach, we distributed the manure applied to soil (calculated in Eqs. (56) and (58) from both FAOSTAT and Einarsson *et al*.^21^ data) according to the country-level proportion of manure applied to cropland and pasture derived from Einarsson *et al*.^21^. The latter study^21^ allocated stored manure to cropland and pasture using country-specific expert estimates of the share of manure applied to cropland and pasture based on national statistics collected for different animal categories for given countries. We derived country-level, annual ratio of manure applied to cropland (and manure applied to pasture) to total manure applied to soil. We then adjusted the gridded amounts of manure applied to cropland and pasture (Eqs. (60) and (62), respectively) to comply with Einarsson *et al*.^21^ estimated ratios.

Overall, by utilizing two different input data sources of manure applied to soil and left on pasture (FAOSTAT and Einarsson *et al*.^21^) and two different methodologies to distribute manure applied to soil between croplands and pastures, we reconstructed four different estimates of gridded manure in our database.

#### Atmospheric deposition

Both reduced and oxidized forms of N from the atmosphere fall back to terrestrial and aquatic ecosystem through either wet deposition (i.e, by precipitation or snow) or dry deposition by process termed N deposition^52^. Atmospheric N deposition in this study was quantified using the National Center for Atmospheric Research (NCAR), Chemistry-Climate Model Initiative (CCMI) N-deposition dataset, which is part of the input datasets for Model Inter comparison Projects (input4MIPS)^53^. The dataset is given at monthly time step from 1850 to 2014 at a spatial resolution of 1.9° latitude by 2.5° longitude. We downscaled the data to the required grid cell size of 5 arcmin resolution using nearest neighbor interpolation and also aggregated to annual time step. To get N deposition for cropland, pasture, forest, semi-natural vegetation, non-vegetation and urban land use, we multiplied the total N deposition with the proportion of respective land area per grid cell.

#### Biological nitrogen fixation (BNF)

BNF is a natural process of transforming atmospheric non-reactive nitrogen (*N*_2_) to its reactive form (ammonia (*NH*_3_) or nitrates) through microbial activity^54,55^. In this study, N fixation over cropland, pasture, forest and semi-natural vegetation (*BNF*_*cr*_, *BNF*_*past*_, *BNF*_*For*_ and *BNF*_*NatVeg*_, respectively) was derived by multiplying the respective areas (${C}_{{A}_{{\rm{crops}}}}$, ${C}_{{A}_{{\rm{past}}}}$, *A*_For_ and *A*_NatVeg_ (*ha*), respectively) with their N fixation rates (*BNF*_*crops-Rate*_, *BNF*_*past-Rate*_, *BNF*_*For-Rate*_ and *BNF*_*NatVeg-Rate*_ (*kg ha*^−1^ *yr*^−1^), respectively); as summarised in Eqs. (63–66).

63 $$BN{F}_{cr}\left(i,{y}_{1850\mbox{--}2019}\right)=\mathop{\sum }\limits_{i=1}^{{n}_{c}}\left({C}_{{A}_{{\rm{crops}}}}\left(i,c,{y}_{1850\mbox{--}2019}\right)\times BN{F}_{crops \mbox{-} Rate}(c)\right)$$

64 $$BN{F}_{past}\left(i,{y}_{1850\mbox{--}2019}\right)={C}_{{A}_{{\rm{past}}}}\left(i,{y}_{1850\mbox{--}2019}\right)\times BN{F}_{past{\rm{ \mbox{-} }}Rate}$$

65 $$BN{F}_{For}\left(i,{y}_{1850\mbox{--}2019}\right)={A}_{{\rm{For}}}\left(i,{y}_{1850\mbox{--}2019}\right)\times BN{F}_{For{\rm{ \mbox{-} }}Rate}$$

66 $$BN{F}_{NatVeg}\left(i,{y}_{1850\mbox{--}2019}\right)={A}_{{\rm{NatVeg}}}\left(i,{y}_{1850\mbox{--}2019}\right)\times BN{F}_{NatVeg{\rm{ \mbox{-} }}Rate}$$

N fixation rates are derived for different crops and land use categories from previous studies^8,21,54^. For fodder crops, we calculated the N fixation rates using the fodder crop production and fixation dataset of Einarsson *et al*.^21^ available at country-level during 1961–2019. The N fixation for the fodder crops in Einarsson *et al*.^21^ was estimated assuming a linear relationship between the crop yield and N fixed by the crops following the approach of Lassaletta *et al*.^47^. We first derived the N fixation rates (*BNF*_*fodder-Rate*_ (*kg kg*^−1^ of crop product)) for the given fodder crop by dividing the values for the N fixation of fodder crops (*BNF*_*fodder*_ (*kg yr*^−1^)) with their respective production (*Pro*_*fodder*_ (*kg yr*^−1^)) as given in Eq. (67). The derived N fixation rates were then multiplied with the fodder crop production during 1850–2019 to get the N fixation by fodder crops (*BNF*_*fodder*_ (*kg yr*^−1^)) as expressed in Eq. (68).

67 $$BN{F}_{fodder \mbox{-} Rate}(c)=\frac{BN{F}_{fodder}(c)}{Pr{o}_{fodder}(c)}$$

68 $$BN{F}_{fodder}\left(i,c,{y}_{1850\mbox{--}2019}\right)=Pr{o}_{fodder}\left(i,c,{y}_{1850\mbox{--}2019}\right)\times BN{F}_{fodder{\rm{ \mbox{-} }}Rate}(c)$$

N fixation rates for pastures was taken as 5 *kg* of N *ha*^−1^ as suggested by Bouwman *et al*.^8^. Moreover, we applied a N fixation rate of 1.77 *kg* *ha*^−1^ *yr*^−1^ for boreal forest (for the forests in Norway, Sweden, Finland, Lithuania, Latvia, Estonia and Russia) and of 16 *kg* *ha*^−1^ *yr*^−1^ for forest in temperate zone (for the forests in all other countries) as given by Cleveland *et al*.^54^. For semi-natural vegetation, we applied the same N fixation rate given for the natural grassland following Cleveland *et al*.^54^ (2.7 *kg* of N *ha*^−1^ *yr*^−1^). N fixation rate for different crops and land types used in this study are listed in Table 2.

### N outputs

The following section provides step wise reconstruction of the N removal from the croplands, pastures and forest. Furthermore, we provide details on the approach used for estimating gridded crop production which is utilized to derive the N removal from harvested crops.

#### N removal from cropland

N removal from cropland (*Rem*_cr_ (*kg yr*^−1^)) was derived as a sum of N removal from all crops calculated by multiplying the crop production (*Pro*_crops_ (*t yr*^−1^)) with crop specific N content (*N*_content_ (*c*) (*kg t*^−1^)); as given in Eq. (69). The coefficients applied to estimate N removal from harvested crops are given in the Table 2.

69 $$Re{m}_{{\rm{cr}}}\left(i,{y}_{1850\mbox{--}2019}\right)=\mathop{\sum }\limits_{i=1}^{{n}_{c}}\left(Pr{o}_{{\rm{crops}}}\left(i,c,{y}_{1850\mbox{--}2019}\right)\times {N}_{{\rm{content}}}(c)\right)$$

Below, we further explained the steps to derive the crop specific annual production during the period 1850–2019.

#### Crop production

We obtained country-level datasets crop production from FAOSTAT for the period 1961–2019^56^. FAOSTAT provides production dataset for 173 crops and excludes dataset for fodder crops. For fodder crops, we derived the dataset from Einarsson *et al*.^21^ that provides estimates for six fodder crops during 1961–2019 at country-level for European countries. For the missing countries and time period in dataset from Einarsson *et al*.^21^, we adapted the same methodology as explained in section “Crop specific harvested area-Fodder crops”. Prior to 1961, we reconstructed the country-level dataset from Bayliss-Smith and Wanmali (1984)^57^ as cited in OWD^58^ which provides wheat yield for the years 1850, 1909, 1934 and 1961 across a few European countries. We generated the annual time series of the wheat yield from OWD (denoted as *Yield*_wheat,OWD_ (*kg ha*^−1^ *yr*^−1^)) for the period 1850–1960 using linear interpolation. The time series of the wheat yield of the countries not included in the OWD dataset was computed as the mean of the wheat yield across the European countries for which data were available. After getting annual wheat yield from 1850 to 1960, we derived wheat production (*Pro*_wheat_ (*kg yr*^−1^)) which is the product of the wheat yield (*Yield*_wheat,OWD_ (*kg ha*^−1^ *yr*^−1^)) and wheat harvested area (${C}_{{A}_{crops}}$ (*ha*), calculated from Eq. (40).

For the reconstruction of crop production for all other crops (other than wheat) during the time period 1850–1960, we utilized the temporal variability of wheat production. To do so, we multiplied the country-level ratio between the wheat production from OWD (*Pro*_wheat,OWD_) for time period 1850–1960 and the wheat production from FAOSTAT ($Pr{o}_{{{\rm{wheat}}}_{FAO}}$) in the year 1961 by the crop production (*Pro*) of all other crops derived from FAOSTAT (for non-fodder crops) and Einarsson *et al*.^21^ (for fodder crops) in 1961 as given in Eq. (70) (all units are in *kg yr*^−1^)).

70 $$Pr{o}_{{\rm{crops}}}\left(u,c,{y}_{1850\mbox{--}1960}\right)=\frac{Pr{o}_{{\rm{wheat}},{\rm{OWD}}}\left(u,c,{y}_{1850\mbox{--}1960}\right)}{Pr{o}_{{{\rm{wheat}}}_{FAO}}\left(u,c,{y}_{1961}\right)}\times Pro\left(u,c,{y}_{1961}\right)$$

We downscaled the country-level crop productions (*Pro*_crops_) using the gridded database of crop production from Monfreda *et al*.^35^ ($Pr{o}_{{{\rm{crops}}}_{Monfreda}}$), as given in Eq. (71) (all units are in *kg yr*^−1^). Monfreda *et al*.^35^ provides crop production for 175 different crops, including both fodder and non-fodder crops, globally at 5 arcmin spatial resolution for the year around 2000. As depicted in Eq. (71) below, this normalisation approach maintained the spatial heterogeneity and consistency in crop production estimates while taking into their respective annual trajectories at a country-level.

71 $$Pr{o}_{{\rm{crops}}}\left(i,c,{y}_{1850\mbox{--}2019}\right)=\frac{Pr{o}_{{{\rm{crops}}}_{Monfreda}}\left(i,c,{y}_{2000}\right)}{{\sum }_{i=1}^{{n}_{u}}Pr{o}_{{{\rm{crops}}}_{Monfreda}}\left(i,c,{y}_{2000}\right)}\times Pr{o}_{{\rm{crops}}}\left(u,c,{y}_{1850\mbox{--}2019}\right)$$

Here, *u* refers to a given country and *n*_*u*_ the total number of grid cells within a country.

#### N removal from pasture

We estimated N removal from pastures (*Rem*_past_ (*kg ha*^−1^ *yr*^−1^)) as harvested and grazed grass following a method proposed in previous studies^8,59^. The method is chiefly based on a nitrogen use efficiency (NUE) in which N removal from pastures is derived by multiplying the N removal coefficient ${C}_{Re{m}_{{\rm{past}}}}$ (-) with the N inputs to pastures (*Inp*_*past*_ (*kg ha*^−1^ *yr*^−1^)) adjusted for the losses of N after manure application to the soil (*N*_*losses*_ (%)) as given in Eq. (72):

72 $$Re{m}_{{\rm{past}}}\left(i,{y}_{1850\mbox{--}2019}\right)={C}_{Re{m}_{{\rm{past}}}}\times \left(In{p}_{past}\left(i,{y}_{1850\mbox{--}2019}\right.\right)-\left({N}_{losses}\times Ma{n}_{past}\left(i,{y}_{1850\mbox{--}2019}\right)\right)$$

The N removal coefficient and N losses for the animal manure are varying among studies^8,59^, and thus are highly uncertain. To account for these uncertainties, we considered two scenarios. In the first scenario, we assumed a value of 0.6 for ${C}_{Re{m}_{{\rm{past}}}}$ based on Bouwman *et al*.^8^ and a value of 0.2 for *N*_*losses*_ based on FAOSTAT. In the second scenario, we assumed a ${C}_{Re{m}_{{\rm{past}}}}$ value of 0.4 and 0.5 for the countries located in Eastern and Western Europe, respectively, based on NUE values reported by Kaltenegger *et al*.^60^ and 0 for *N*_*losses*_.

#### N removal from forest

N removal from forest (*Rem*_for_ (*kg ha*^−1^ *yr*^−1^)) was estimated by adapting the method from Chang *et al*.^61^, which assumes that N atmospheric deposition has a N fertilization effect and leads to an increase in woody biomass. To account for this aspect, we multiplied the atmospheric N deposition (*DEP*_for_ (*kg ha*^−1^ *yr*^−1^)) by the rate of forest N removal (${C}_{Re{m}_{{\rm{For}}}}$ (-)) as given in Eq. (73).

73 $$Re{m}_{{\rm{For}}}\left(i,{y}_{1850\mbox{--}2019}\right)={C}_{Re{m}_{{\rm{For}}}}\times DE{P}_{{\rm{For}}}\left(i,{y}_{1850\mbox{--}2019}\right)$$

We selected a value of 0.02 for ${C}_{Re{m}_{{\rm{For}}}}$ based on the values suggested by Chang *et al*.^61^.

### N surplus across Europe (1850–2019)

**Fig. 2:**
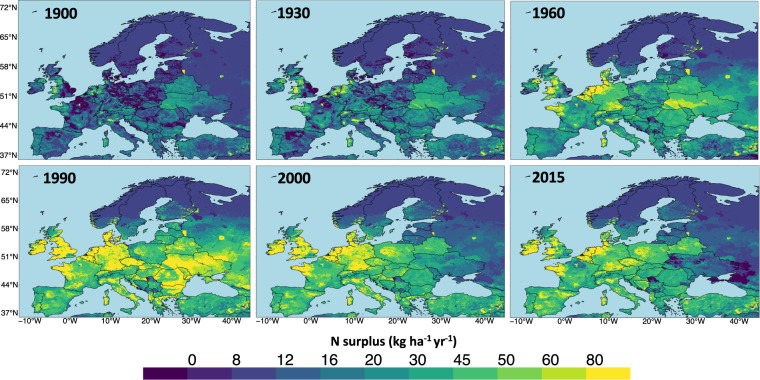
Snapshots of N surplus (*kg ha*^−1^ of grid area *yr*^−1^) across Europe. The figure shows the annual spatial variation in N surplus given as the mean of our 16 N surplus estimates for the selected years.

**Fig. 3:**
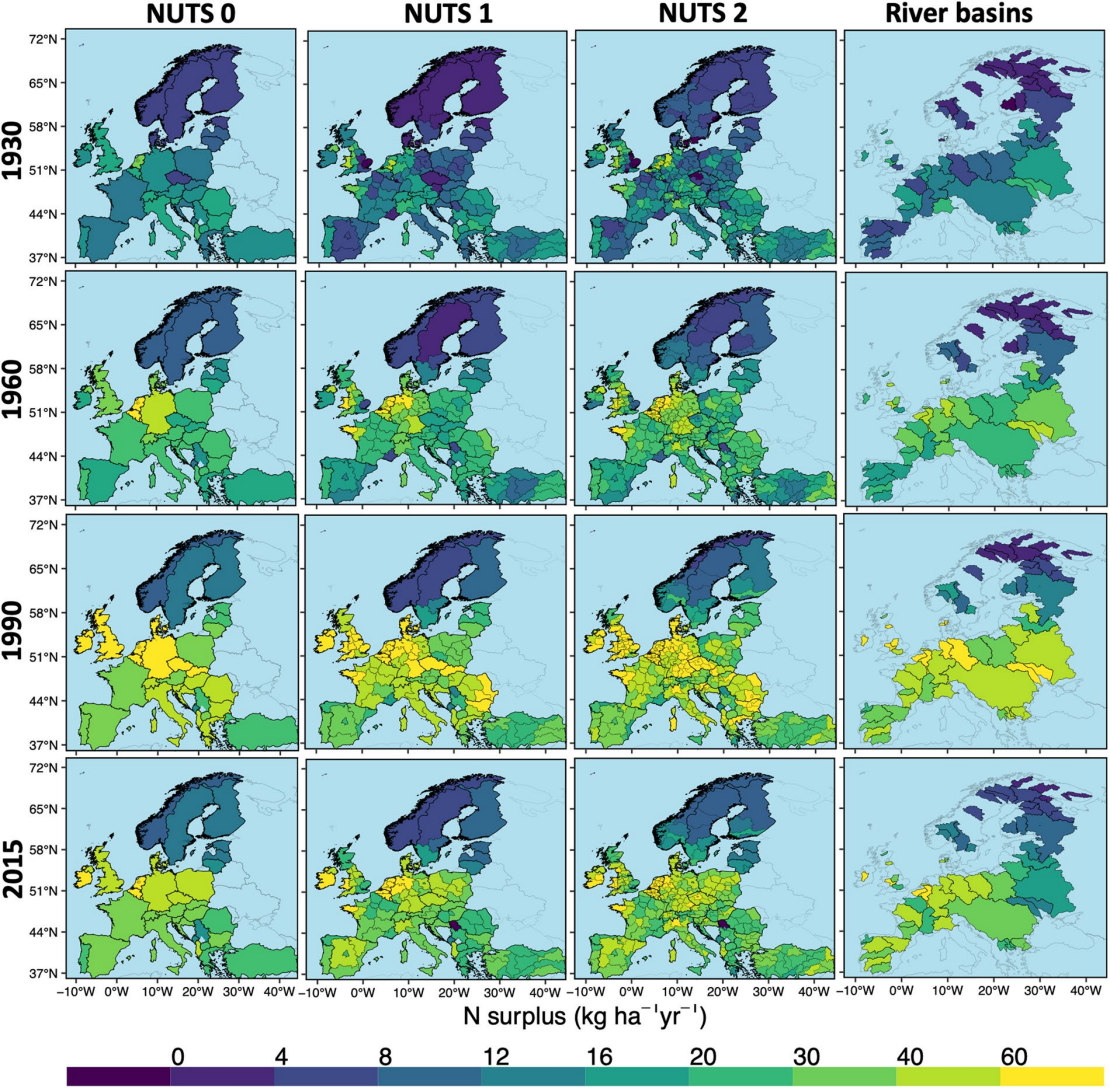
Total N surplus (*kg ha*^−1^ of physical area *yr*^−1^) at multiple spatial levels for four years (1930, 1960, 1990, 2015). N surplus is given as the mean of our 16 N surplus estimates. NUTS: Nomenclature of Territorial units for statistics.

**Fig. 4:**
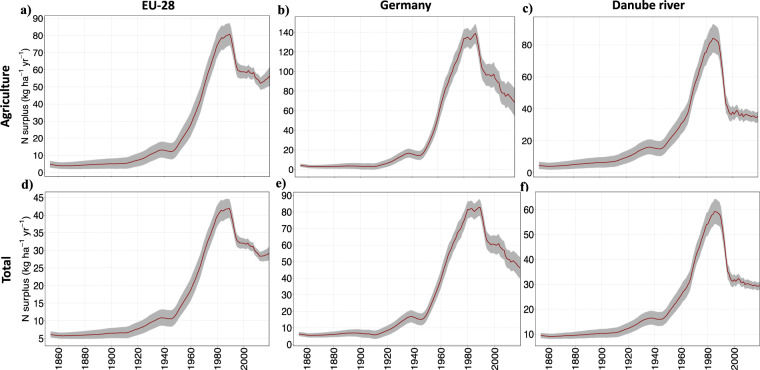
Agricultural N surplus (*kg ha*^−1^ of agricultural area *yr*^−1^) and total N surplus (kg ha^−1^ of physical area yr^−1^) for EU-28, Germany and the Danube river basin (5 years moving average during 1850–2019). The grey color ribbon in each panel shows the ranges (minimum and maximum values) of the 16 N surplus estimates reconstructed in this study, whereas the average value is presented by a red line. **(a**–**c)** Agricultural N surplus for EU-28, Germany and Danube river, **(d**–**f)** Total N surplus for EU-28, Germany and Danube river.

We constructed 16 N surplus estimates at the spatial resolution of 5 arcmin (1/12°) across Europe, while accounting for the uncertainties in major components of N surplus. Specifically, we obtained two datasets for fertilizer, four datasets for animal manure and two datasets for N removal from pastures. Here, we present an average value of the 16 N surplus estimates during the time period of 1850–2019 in Figs. 2 and 3 and we show the uncertainties in Fig. 4.

The spatio-temporal variability in our total N surplus at gridded level is presented in Fig. 2. Here, we present the snapshot for the year 1900, 1930, 1960, 1990, 2000 and 2015 for the illustration. We observed a clear latitudinal pattern with lower values of the N surplus in northern Europe, higher values at mid-latitudes in particular in Western and Central Europe, to moderate value in southern Europe. Furthermore, N surplus showed large temporal changes during the period of 1850–2019 in most places, apart from northern Europe where its value remained relatively low and stable. This reflects the importance of having a long-term dataset of N surplus that allows quantification of these temporal developments. Up to 1900, N surplus in the majority of the grid cells did not exceed 20 *kg* *ha*^−1^ *yr*^−1^. N surplus increased by a factor of 2–3 in around 70% of the grid cells during the time period of 1930–1990 (from around 20 *kg* *ha*^−1^ *yr*^−1^ in 1930 to values in the range 40–60 *kg* *ha*^−1^ *yr*^−1^ in 1990). In most of the grid cells of the industrialized European countries, N surplus reached its higher value around 1990 and declined in the subsequent time period. This decline was particularly apparent in countries such as Denmark, Germany, Latvia, Lithuanian, Estonia, Belarus, Ukraine etc. Conversely, Spain continued to show an upward trend in the majority of the grid cells during 1960–2000 with a gradual downwards trend afterwards. The gridded N surplus allows to examine the N surplus at sub-country levels, such as the levels of the European “Nomenclature of Territorial units for statistics” (NUTS) classification as depicted in Fig. 3. This figure shows the value of having information at sub-country level, since country-level (NUTS 0) averaging of N surplus can conceal large disparities between the territories. For instance, in 2015, the N surplus at NUTS 0 (country) level for France and the United Kingdom took moderate values (29 and 38 *kg* *ha*^−1^ *yr*^−1^, respectively). However, the NUTS 1 and NUTS 2 (sub-country) level information show that the N surplus was high in part of these countries, with values reaching 76 *kg* *ha*^−1^ *yr*^−1^ in the Brittany region in north-west France and 69 *kg* *ha*^−1^ *yr*^−1^ in Wales in the UK. Our gridded dataset also allows with the flexibility to assess the temporal trajectories of the N surplus for any river basin that may span different countries, as shown in Fig. 3 (right most panel). This is critical because river basins are relevant spatial scale for water quality modelling and land and water management^12,62^. Between 1930 and 1960, N surplus in the majority of the river basins showed a constant increasing pattern, a sharp jump in 1990 and declined afterwards. For instance, in the basin of the Danube river, that flows through much of the Central and Southeastern European countries, N surplus showed around a two-fold growth during 1930–1960 (14–26 *kg* *ha*^−1^ *yr*^−1^), further increased by a factor of 1.9 between 1960–1990 (49 *kg* *ha*^−1^ *yr*^−1^ in 1990), and reduced by around 39% during 1990–2015 (30 *kg* *ha*^−1^ *yr*^−1^ in 2015).

Figure 4 shows that the uncertainty range (between the maximum and minimum values of our 16 N surplus estimates) of agricultural and total N surplus estimates varies over time and across regions. Here for the illustration, we take N-surplus estimates for the scale of EU-28, Germany and Danube river basin. In general, for the agricultural N surplus, we observed that, during 1850–1910, the width of the uncertainty intervals (grey ribbons in Fig. 4a–c) has a similar magnitude to the mean estimate (red lines) for EU-28 and the Danube river and it is even more than twice the mean estimate for some years for Germany. Between 1910–2019, the width of the uncertainty range relative to the mean estimates decreased by a factor of around 7 for both EU-28 and the Danube river basin, i.e. it is equal to around 10–20% of the mean in 2019. For Germany, it took higher values than EU-28 and the Danube river basin for the recent years (around 40% of the mean estimate in 2019). In terms of absolute differences between the maximum and minimum estimates, we observed that the width of the uncertainty interval (grey ribbons) gradually increased during 1850–1950 (3–9 *kg* *ha*^−1^ *yr*^−1^) for EU-28, Germany and the Danube river basin (Fig. 4a–c). Between 1950–1990, the values for the Danube river basin kept on increasing (up to almost 15 *kg* *ha*^−1^ *yr*^−1^ in the 1980s), EU-28 showed a more stable pattern (10–13 *kg* *ha*^−1^ *yr*^−1^). After 1990, the uncertainty bound (ribbons) exhibited first a gradual decrease in thickness for EU-28 and the Danube river basin (down to around 5 *kg* *ha*^−1^ *yr*^−1^ in the 2000s) and a slight increase was seen in the recent years for EU-28. Comparatively, N surplus for Germany has a much larger uncertainty interval during 1950–2019 that keeps widening in time to reach a width of around 28 *kg* *ha*^−1^ *yr*^−1^ after 2015. We noticed a similar pattern in the uncertainty range over time for the total N surplus for EU28, Germany and the Danube river basin (Fig. 4d–f). These analyses highlight the value of our ensemble N surplus dataset, emphasising the uncertainty in N surplus estimates.

## Data Records

The dataset comprises 16 N surplus estimates in the Network Common Data Form (NetCDF) file format. Each NetCDF file contains an annual variable representing N surplus with the unit *kg ha*^−1^ of grid area *yr*^−1^ at 5 arcmin (1/12°) resolution for the period 1850–2019. We also provide N surplus estimates at aggregated spatial levels (i.e. NUTS 1, NUTS 2 and selected river basins covering in and around the areas of Europe) in comma-separated value (.csv) formats. The datasets are archived at the Zenodo repository^63^. The description of the files provided in data repository is given below:

- 16 files termed as “N_sur_total_kg_ha_grid_1850_2019_method_xx_v1.0” for gridded N surplus data in NetCDF format. Here, xx presents dataset from method 1 to method 16 and each of them contains 170 years (1850–2019) of gridded N surplus.
- 3 files termed as “N_sur_total_kg_ha_NUTS_xx” for N surplus at spatial aggregated levels in .csv format. Here, xx presents NUTS 1, 2 and 3 and each of them contains mean and standard deviation of 16 N surplus estimates.
- 1 file termed as “N_sur_total_kg_ha_river_basin” for N surplus at selected river basin levels in .csv format. This file contains mean and standard deviation of 16 N surplus estimates.

Additionally, readme files are provided for the NUTS and river basins ID’s.

## Technical Evaluation

To check the spatio-temporal plausibility and consistency of our N surplus dataset, we compared our 16 N surplus estimates with available datasets (see Table 3), due to a lack of direct observations of N surplus. First, we used the datasets of the statistical office of the European Union (Eurostat)^19^ and Zhang *et al*.^5^, available at country-level across Europe for the period 1990–2019 and 1961–2015, respectively. The dataset of Eurostat^19^ is a reference dataset for Europe that consistently provides N land budgets for agricultural areas across 37 European countries. Zhang *et al*.^5^ provides different components of the N surplus for croplands by synthesising information of 13 different N datasets; and as such it has been proposed as a benchmark estimate^5^. To check for further consistency, we compared our N-budget with country specific estimates for Denmark^22^ (1990–2010), the United Kingdom^23^ (1990, 1995 and 2000–2018), France^24^ (1940–2010), Poland^25^(1960–2000) and Germany^26,64^,(1950–2017), while results for 25 European countries were additionally compared to estimates given by Leip *et al*.^20^ for the period of 2001–2003. For most of these studies, we could only extract information at country-level. An exception is the German dataset for which we could derive NUTS 1 (state) level data for the period 1950–2015 combining the two datasets of Behrendt *et al*.^64^ and Häussermann *et al*.^26^ (for methodological details we refer to Ebeling *et al*.^65^).

**Table 3:** Existing datasets used for technical evaluation and comparison in this study.

Datasets	Budget type	Land type	Time period	Spatial information	References
Eurostat	N land budget	Agriculture	1990–2019 (kg ha^−1^ yr^−1^ of agriculture area), 2010–2014 (tonne)	Country (Europe)	^19^
Zhang *et al*.^5^	N soil surface budget	Cropland	1961–2015	Country (Global)	^5^
Hutchings *et al*.^22^	N soil surface budget	Agriculture	1990–2010	Country (DK)	^22^
Defra^23^	N soil surface budget	Agriculture	1990, 1995 and 2000 to 2020	Country (UK)	^23^
Poisvert *et al*.^24^	N soil surface budget	Agriculture	1940–2010	Country (FR)	^24^
Eriksson *et al*.^25^	N soil surface budget	Agriculture	1960–2000	Country (PO)	^25^
Häussermann *et al*.^26^	N soil surface budget	Agriculture	1995–2017	Country (DE)	^26^
Behrendt *et al*.^64^	N soil surface budget	Agriculture	1950–1998	NUTS 1 (DE)	^64^
Leip *et al*.^20^	N soil surface budget	Agriculture	2001–2003	Country (Europe)	^20^

At continental-level, the uncertainty intervals of our N surplus (assessed as one standard deviation around the mean of the 16 estimates) captured the values derived from Eurostat^19^ and Zhang *et al*.^5^. Specifically, our average cropland N surplus for 20 countries over the period 1961–2015 is equal to 7.86 ± 1.18 Tg of N, whereas Zhang *et al*.^5^ provides a value of 9.2 Tg of N. Our average agricultural N budget for EU-28 over the period 2010–2014 is equal to 8.9 ± 0.75 Tg of N, while the value calculated from Eurostat is 8.88 Tg of N. Moreover, with respect to the Leip *et al*.^20^ study which reported an average N surplus of 59 *kg* *ha*^−1^ *yr*^−1^ for the period 2001–2003 for EU-27, our dataset show a reasonably good match with N surplus values of around 60 ± 4 *kg* *ha*^−1^ *yr*^−1^ for the same time period. With respect to country specific study, Häussermann *et al*.^26^ reported an average value of 77 *kg* *ha*^−1^ *yr*^−1^ for N soil surface budget for Germany during 2015–2017, which is also included in our uncertainty bound of 70 ± 9 *kg* *ha*^−1^ *yr*^−1^. N surplus estimates for both France (47 ± 4 *kg* *ha*^−1^ *yr*^−1^, average over 1940–2010) and Poland (55 ± 4 *kg* *ha*^−1^ *yr*^−1^ an average of 1960–2000) are slightly higher in our study compared to the values found in previous studies that are equal to 37 *kg* *ha*^−1^ *yr*^−1^ ^24^ and 41 *kg* *ha*^−1^ *yr*^−1^ ^25^, respectively.

**Fig. 5:**
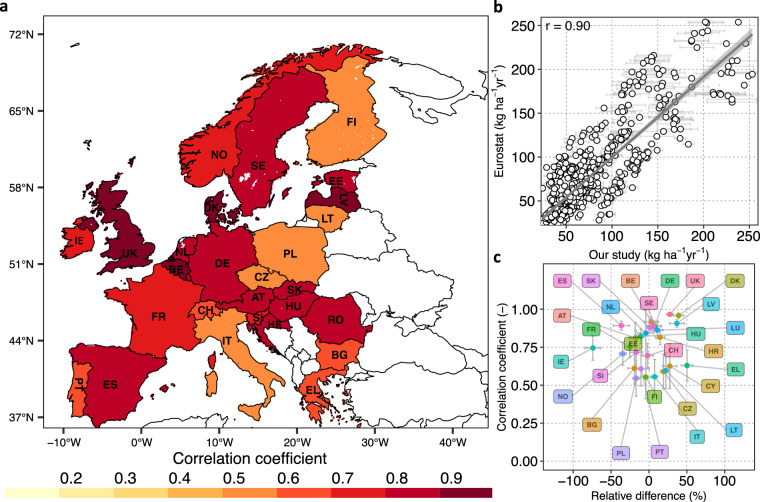
Country level comparison between N surplus for agriculture soils estimated by Eurostat^19^ and this study for the period 1990–2019. The circles in panels (**b)** and (**c)** denote the average of the 16 N surplus estimates reconstructed in this study, whereas the bars show the standard deviation. **(a)** Pearson correlations coefficients (r) values for every country. Countries with white color are excluded from the comparison because they are not part of the Eurostat dataset. (**b)** Linear fit between the N surplus values for all countries and all years in the two studies: x-axis shows the N surplus calculated in this study and y-axis presents the N surplus given by Eurostat, **(c)** Relative difference (defined in Eq. 74) in N surplus in this study with respect to Eurostat against correlation coefficient for each country. Romania (RO) is not presented in this plot as it is an outlier with a value of −111% for mean relative difference and a SD of 35% around the mean. The correlation for Romania is however high (0.9 ± 0.03).

**Fig. 6:**
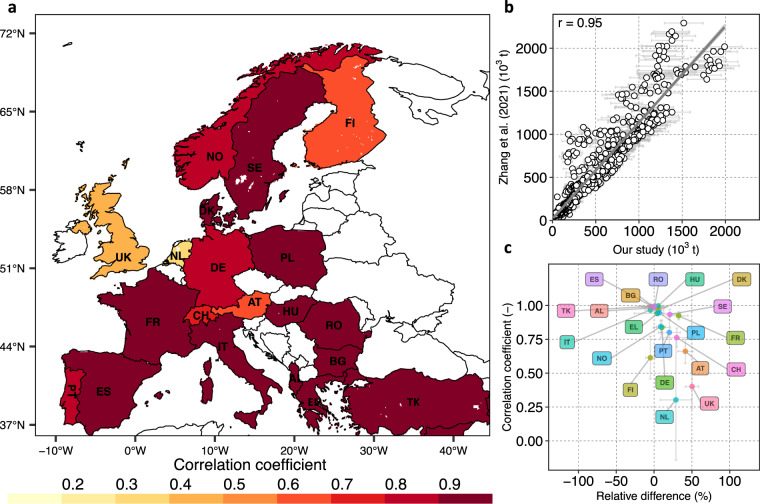
Country level comparison between N surplus for the cropland estimated by Zhang *et al*.^5^ and this study for the period 1961–2015. The circles in panel (**b**) and (**c**) denote the average of the 16 N surplus estimates reconstructed in this study, whereas the bars show the standard deviation. (**a**) Pearson correlations coefficients (r) values for every country. Countries with white color are excluded from the comparison because they are not part of the Zhang *et al*.^5^ dataset, (**b**) Linear fit between the N surplus values for all countries and all years in the two studies: x-axis shows the N surplus calculated in this study and y-axis presents the N surplus given by Zhang *et al*.^5^(**c**) Relative difference (defined in Eq. 74) in N surplus in this study with respect to Zhang *et al*.^5^ against correlation coefficient for each country.

To determine the differences in the average N surplus from this study with respect to Eurostat data (respectively Zhang *et al*.^5^), we calculated the country-wise relative difference *Diff* (%), as given in Eq. 74:

74 $$Diff=\left(\frac{Referenc{e}_{Nsur}-Stud{y}_{Nsur}}{Referenc{e}_{Nsur}}\right)\times 100$$

where *Reference*_*Nsur*_ denotes the average N budget given by Eurostat during the time period 1990–2019 (in *kg ha*^−1^ of agricultural area *yr*^−1^) (respectively Zhang *et al*.^5^ between 1961–2015 in *tonne*) and *Study*_*Nsur*_ denotes one of our 16 N surplus estimates averaged over the relevant time period. We present here the mean and standard deviation (SD) for the 16 value of *Diff* calculated for each of our 16 N surplus datasets. We found that, in about 70% of the countries, the mean *Diff* between our estimates and Eurostat was contained in the range ± 30% (see Fig. 5c). Large values of the mean *Diff* (exceeding ± 50%) were observed for some countries, namely Romania (RO; *Diff* = −111), Ireland (IE; *Diff* = −63) and Greece (EL; *Diff* = 50). We note that these differences in N surplus between Eurostat and our estimates could occur because Eurostat unlike our estimate accounts for volatilization losses during manure management and storage. With respect to Zhang *et al*.^5^, 85% of the countries show a mean *Diff* in the range ± 30% (see Fig. 6c), and all countries have a mean *Diff* within the range ± 50%. In particular, higher values (in the range 32–50%) are found for the United Kingdom (UK), France (FR) and Austria (AT). Importantly, when considering the entire interval of *Diff* (across our 16 estimates) beyond the mean value only, we found a better correspondence between Eurostat^19^ and Zhang *et al*.^5^ datasets and ours. In fact, we observed that the interval contains values in the range ± 30% for 80% of the countries with respect to Eurostat^19^ data and for 95% of countries with respect to Zhang *et al*.^5^ data. Nonetheless, in general, the bounds of our 16 values of *Diff* are reasonably narrow for both Eurostat^19^ and Zhang *et al*.^5^. In particular, we observed that the SD of *Diff* is lower than or equal to around 11% for all countries (except for Romania) with respect to Eurostat^19^ and for 70% of the countries with respect to Zhang *et al*.^5^ data.

To assess the consistency of the temporal dynamics between the N budget from this study and available datasets, we computed a correlation coefficient (*r*). Here, we present the mean and standard deviation of 16 *r* values derived for each of our 16 N surplus datasets. Overall, we found a strong, positive correlation between estimates of this study and Eurostat^19^ during 1990–2015 (*r* = 0.90 ± 0.03; see Fig. 5b). In three-quarters of the countries, we observed high mean *r* values in the range 0.6–0.9, and none of the countries have a mean *r* value below 0.5 (Fig. 5a). The dynamics of our cropland N budgets also shows positive and strong correlation with Zhang *et al*.^5^ during 1961–2015 (r = 0.95 ± 0.02); see Fig. 6b). In around three-quarters of the countries, there is a very good consistency between our estimates and Zhang *et al*.^5^, with mean *r* values in the range 0.8–0.99 (Fig. 6a). In the Netherlands (NL), Finland (FI) and the United Kingdom (UK), we noticed lower mean values of *r* (in the range 0.3–0.6). However, the SD of the *r* values across the 16 N surplus estimates is large for these three countries. This results in values of the upper bound of *r* (one SD above the mean value) equal to 0.75, 0.56 and 0.77 for NL, FI and UK, respectively, which is much higher than the mean *r* value. This means, that the temporal dynamics of some of our N surplus estimates are reasonably consistent with Zhang *et al*.^5^ data for these three countries. Overall for most countries, we found that the spread of our 16 *r* values is reasonably narrow for both Eurostat^19^ and Zhang *et al*.^5^. This reflects that the temporal dynamics across our 16 N surplus estimates are similar. Specifically, the SD of *r* is lower than 0.10 for 80% of the countries with respect to Eurostat^19^ and for 85% of the countries with respect to Zhang *et al*.^5^.

**Fig. 7:**
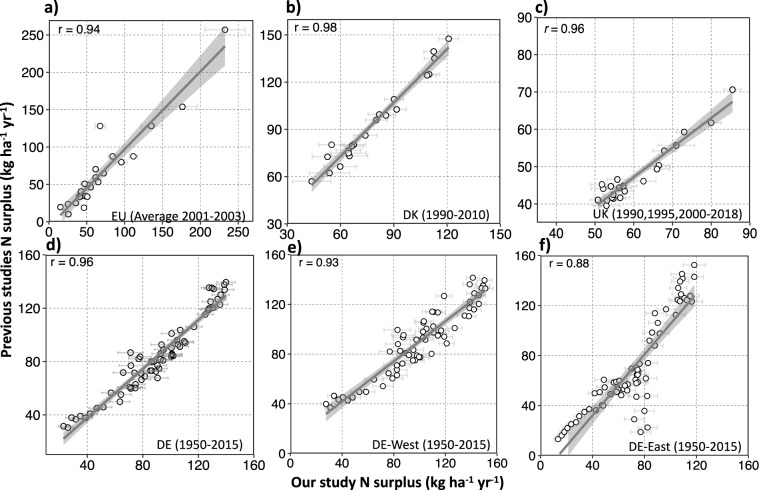
Scatter plots between the N surplus for agricultural soil from various previous studies and our study for different countries and corresponding linear fits. The circles in each panel denote the average of 16 N surplus reconstructed in this study, whereas the bars show the standard deviation. **(a)** European countries during 2001–2003 (each point in this plot represent one country), **(b)** Denmark for each year in 1990–2010, **(c)** United Kingdom (UK) for 1990, 1995 and each year in 2000–2018, **(d)** Germany (DE) for each year in 1950–2015, **(e)** Aggregated values over the states of the former West Germany (DE-West) for each year in 1950–2015, **(f)** Aggregated values over the states of the former East Germany (DE-East) for each year in 1950–2015.

Similarly, we observed that our N budget is strongly and positively correlated with estimates for EU countries (*r* = 0.94 ± 0.01) during 2001–2003 compared to Leip *et al*.^20^ estimates (see Fig. 7a), Denmark (DK) (*r* = 0.98 ± 0.01) between 1990–2010 (see Fig. 7b), and United Kingdom (UK) (*r* = 0.96 ± 0.01) for the time period 1990–2009 (Fig. 7c) and Germany (DE) (*r* = 0.96 ± 0.01) between 1950–2015 (Fig. 7d). Furthermore, for Germany we compared our estimates at a sub-country (NUTS 1) level based on the harmonized dataset of Behrendt *et al*.^64^ and Häussermann *et al*.^26^ for the years 1950–2015. We examined the results aggregated over the states of the former West and East Germany and found *r* values of 0.93 ± 0.04 and 0.88 ± 0.04, respectively, showing a strong and positive association between the sub-country scale German dataset and ours (see Fig. 7e,f). We also observe for some years much lower values of the N surplus for East Germany in the dataset^26,64^ compared to ours. The two datasets show different temporal dynamics around the year of the German reunification (1990), when agricultural activities were disrupted.

Overall, we found that our 16 estimates of N surplus at continental and country-level captured reasonably well other existing estimates provided by previous studies in terms of their spatio-temporal pattern and relative differences. However, we found some differences in N surplus estimates, which emphasizes the need for a more comprehensive characterization of the uncertainties in the N surplus in the future, to further improve the N surplus estimation. In fact, N surplus datasets are the result of a modeling process involving the use of uncertain underlying data and numerous uncertain methodological choices. Only part of these uncertainties were considered in our study, while we discuss in the following other sources of uncertainty that could explain the differences between our estimates and previous studies. First, the input data sources of different components of the N surplus such as fertilizer, animal manure, atmospheric deposition differ among studies, which could lead to differences in estimated amounts among studies. For example, regarding fertilizers, the database of IFA^66^ relies on sales/trades data that mainly come from fertilizer companies of their member countries, FAOSTAT^41^ collects information officially from its member countries via questionnaire and Eurostat^19^ obtained consumption amount from country specific statistics (i.e. data based on trade, production of fertilizer and surveys) and from UNFCCC (United Nations Framework Convention on Climate Change) for the missing countries^67^. Second, studies made various assumptions regarding the amount of fertilizer and animal manure applied to cropland and pastures. For example, many studies documented in Zhang *et al*.^5^ did not exclude the fertilizer used on pastures to assess cropland application. Thus, there is a large uncertainty in the spatial distribution of applied N. Moreover, differences in N budget can largely be attributed to different coefficients used by different studies for estimating for instance N content in harvested crops, N fixation and volatilization losses in animal manure^28^. Another plausible cause of uncertainty in N budget is the different crop types considered in the studies, especially fodder crops^27^.

Despite the above-mentioned challenges, our dataset is a step forward since it provides 16 uncertainty estimates in major components of the N surplus, while previous datasets^5,19,21–23,25,26^ typically do not report uncertainties in N surplus. The findings of the comparison of our N surplus estimates with previous datasets highlight the need to further characterize the uncertainties in future versions of the N surplus dataset.

## Usage Notes

This study makes available annual estimates of N surplus from 1850 to 2019 at gridded resolution across Europe. We accounted for uncertainties due to different underlying data and methodological choices in components of N surplus that are reported to have considerable uncertainties^5^, namely N inputs from fertilizer and manure, and N removal from pastures. Our uncertainty ensemble was able to generally capture well country-level N surplus estimates available from previous studies/reports/databases (see Section “Technical evaluation”). We do acknowledge that our dataset also does not include all sources of uncertainties, and we discussed further uncertain data and choices that are worth investigating in future versions of N surplus estimates (Section “Technical evaluation”). In particular, further considerations can be given to better inform about disaggregation schemes and their associated uncertainties. In this study, we downscaled the country level estimates (e.g. the fertilizer application amounts for the different crop categories in 2014/2015 from IFA^40^) using the spatial variability in land use areas (Ramankutty *et al*.^34^ dataset), and crop specific harvested areas and crop production from the Monfreda *et al*.^35^ dataset. Moreover, the construction of country-level N surplus components before 1961 is based on either global temporal dynamics (e.g. for fertilizers) or proxy information (e.g. for crop yields, we assumed the same dynamic of yield as of wheat). Hence, our database is likely to have less reliability during the time period 1850–1960. Comparatively, we can consider that our estimates have a lower uncertainty and a moderate reliability after 1961, since there is better availability of N surplus components at country level (e.g. estimates of fertilizer and manure from FAOSTAT). Furthermore, we expect that our N surplus data are less uncertain for the recent time period (from the mid-1990s). This is mainly due to the fact that we utilized detailed spatial dataset for the reconstruction of N surplus estimates during this time period (e.g. land use and crop production datasets that are available around 2000).

Therefore, users should be aware that our N surplus data are more reliable for the recent time frame, while past estimates are valuable for informing general trends in the N surplus values. Regarding the spatial scale, we recommend not to use our dataset at gridded level, because of the uncertainties in the spatial disaggregation schemes, but rather at higher aggregation levels, such as country level, the different European socio-economic regions/states/provinces (e.g. NUTS 0, NUTS 1, NUTS 2 levels), and river basin scale (see Section “N budgets across Europe” and Fig. 3) to support different land and water management activities. Importantly, we provide here a consistent methodology using publicly available information for the reconstruction of N surplus over a long time period. Our methodology thus, can be reused to incorporate new underlying datasets and further updates of datasets that we already used, such as a new version of the FAOSTAT database, as they become available.

## Data Availability

We used the RStudio version (1.2.5019) for data processing. The codes for the study are available at GitLab https://git.ufz.de/batool/n_surplus.
